# Supplementary figures and images for: Ultrastructural insights into the microsporidian infection apparatus reveal the kinetics and morphological transitions of polar tube and cargo during host cell invasion
Source: PLoS Biol. 2024 Feb 29;22(2):e3002533. doi: 10.1371/journal.pbio.3002533 (PMC10931468; doi:10.1371/journal.pbio.3002533)

S2 Data File  
Uncropped SDS-PAGE gel used in Fig 4c.

Fig 4c

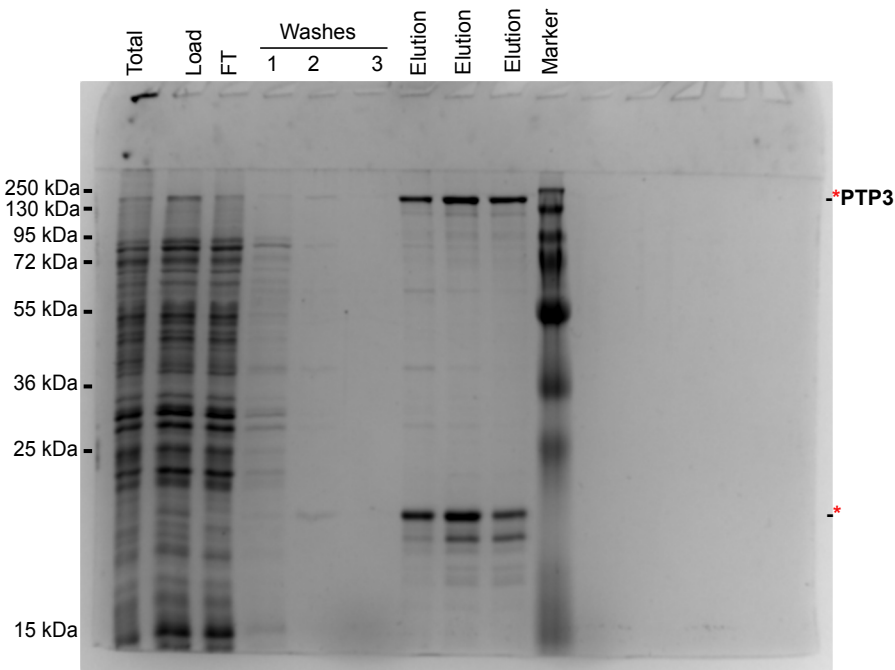

Supplement: S1 Raw Image — (PDF) [file pbio.3002533.s002.pdf]

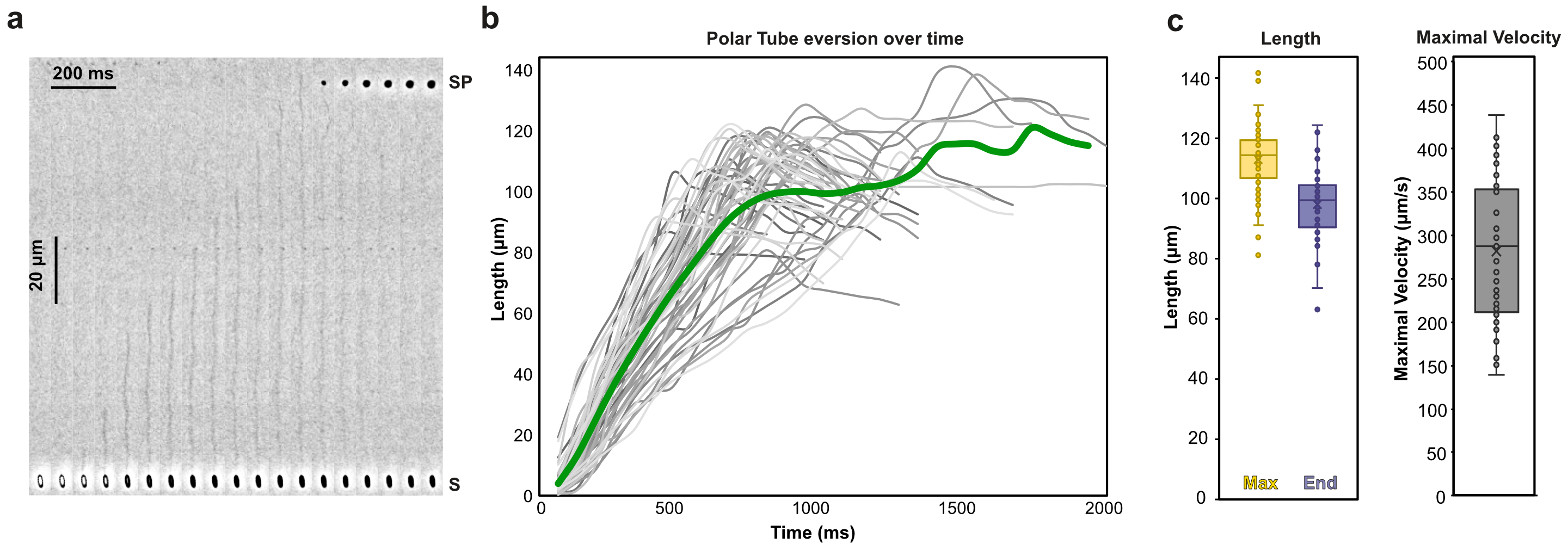

Supplement: S1 Fig — (a) A kymograph obtained via live light microscopy analysis of polar tube firing events from Vairimorpha necatrix. The spore at the bottom of the kymograph is denoted by “S” and the sporoplasm ejected on the distal end is indicated as “SP.” (b) Length over time diagrams of all analyzed polar tube eversion events. The average length over time is colored in green. (c) Bar plots of polar tube maximal length (yellow), length at the end (blue), and maximum velocity distribution (gray). The raw data used to create the plots can be found in the S1 Data. (PNG) [file pbio.3002533.s007.png]

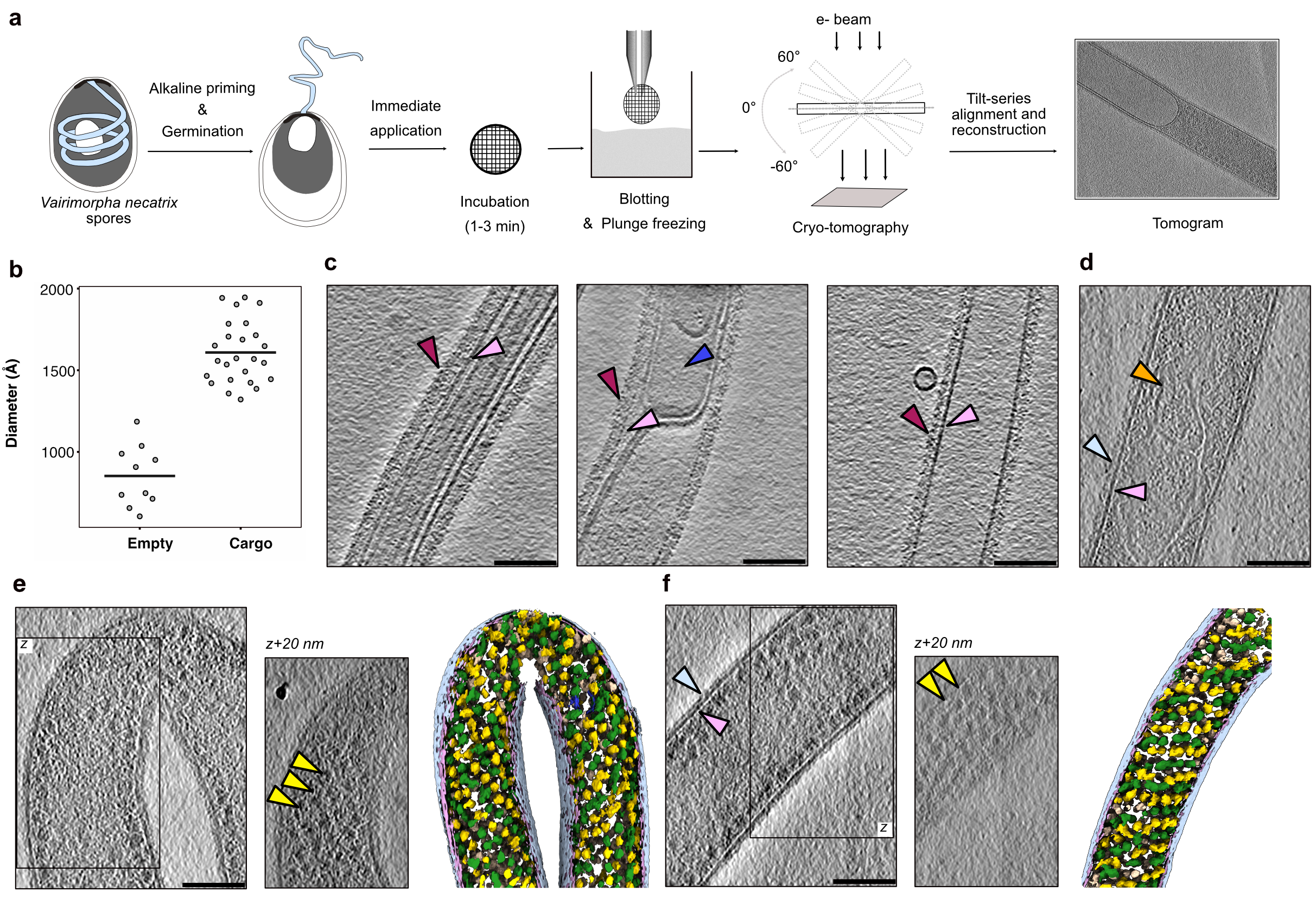

Supplement: S2 Fig — (a) A schematic representation of the methodology for on-grid freezing and collecting tomograms of germinated polar tubes. (b) A graph representing the internal diameter of polar tubes (PTempty and PTcargo) visualized using cryo-ET. Each dot represents 1 tube, and the line represents the mean diameter. The raw data underlying this figure can be found in the S1 Data file. (c) Representative tomograms of PTempty or polar tubes filled with electron-lucent material or completely devoid of cellular cargo. The central section of a tomogram is shown with regions of interest indicated with arrows (magenta for the outer wall, pink for the lipid bilayer, and blue for vesicles). (d–f) Representative tomograms from PTcargo or polar tubes filled with cellular cargo where (e and f) contained ribosome spirals inside tubes. The central section of a tomogram is presented, and regions of interest are indicated with arrows (light blue for the outer tube wall, pink for the lipid bilayer, and yellow for ribosomes). For (e and f), additional views corresponding to the boxed regions and corresponding segmented tomograms are also presented. (PNG) [file pbio.3002533.s008.png]

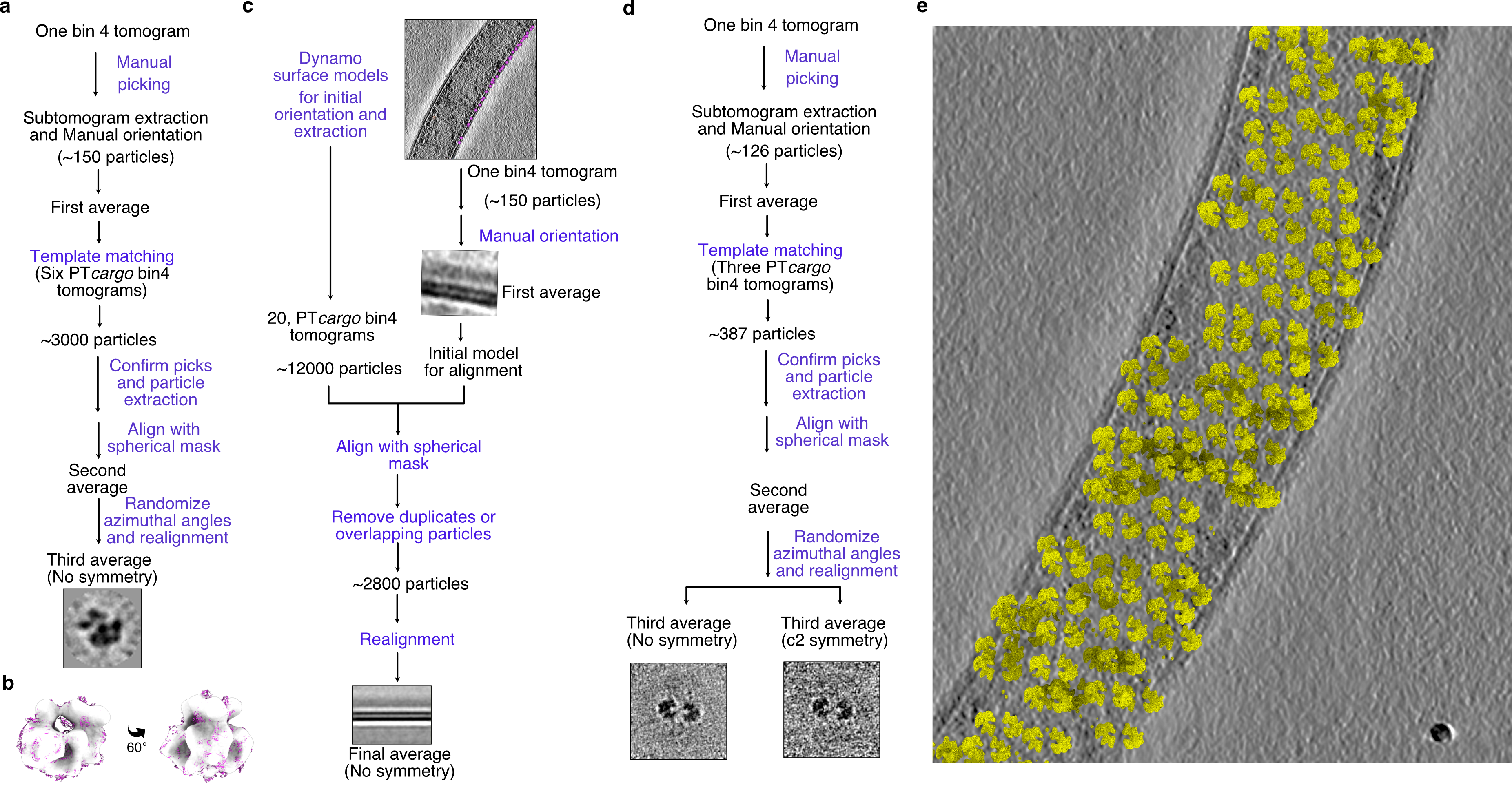

Supplement: S3 Fig — (a) Schematic workflow of the subtomogram averaging procedure to generate the ribosome volume. (b) Two 60°-related views of ribosome reconstruction (transparent white), fitted with the structure of the V. necatrix ribosome (PDB ID: 6RM3, magenta). (c) Schematic workflow of the subtomogram averaging procedure used to create the reconstructions of the segments of polar tube outer layers. The scheme is shown for cargo-filled tubes, and a similar methodology was used for empty tubes. (d) Subtomogram averaging workflow used to reconstruct dimeric ribosomes from clustered particles in sporoplasm-filled tubes. (e) Low-pass filtered subtomogram averages of ribosome dimers placed back into their original location in germinated polar tubes. Averages are shown in yellow, and the central slice of the tomogram slice is shown in gray. (PNG) [file pbio.3002533.s009.png]

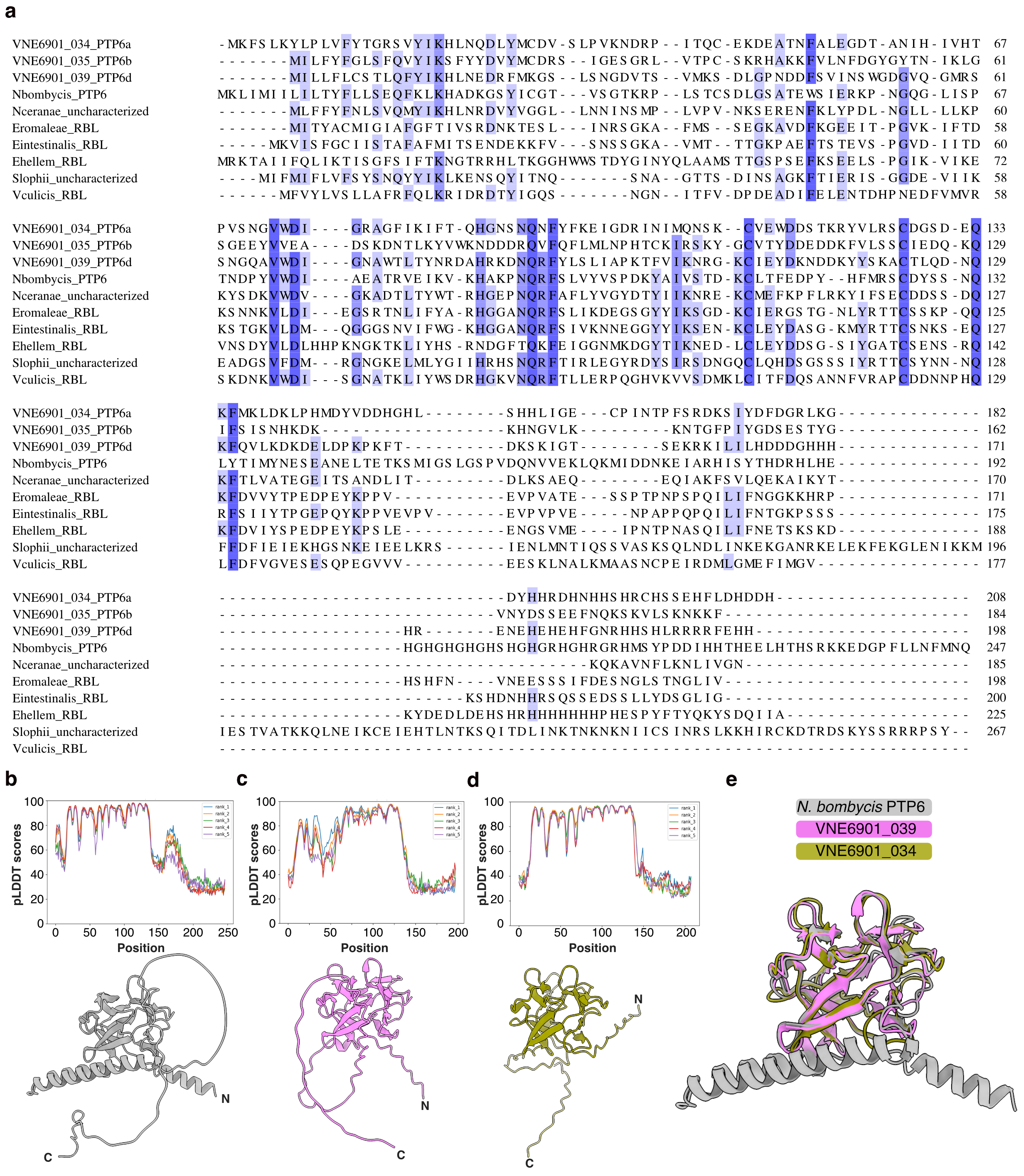

Supplement: S4 Fig — (a) Alignment of VNE6901_039 and VNE6901_034 with PTP6 homologs from Nosema bombycis (R0MBR8_NOSB1), Vavraia culicis (L2GVW3_VAVCU), Nosema ceranae (C4V7Y1_NOSC), Spraguea lophii (S7XK85_SPRLO), Encephalitozoon hellem (I6TKU6_ENCHA), Encephalitozoon romaleae (I7AT09_ENCRO), Encephalitozoon intestinalis (E0S8R2_ENCIT). Protein sequences were retrieved from Uniprot and aligned using Muscle followed by visualization using Jalview. (b–d) Alphafold models, below their corresponding pLDDT scores of the top-ranked predictions, for N. bombycis PTP6 (b), VNE6901_039 (c), and VNE6901_034 (d). (e) Overlay of predicted PTP6 models from (b–d) shown at 90° rotation. Regions predicted with low confidence have been excluded for clarity. (PNG) [file pbio.3002533.s010.png]

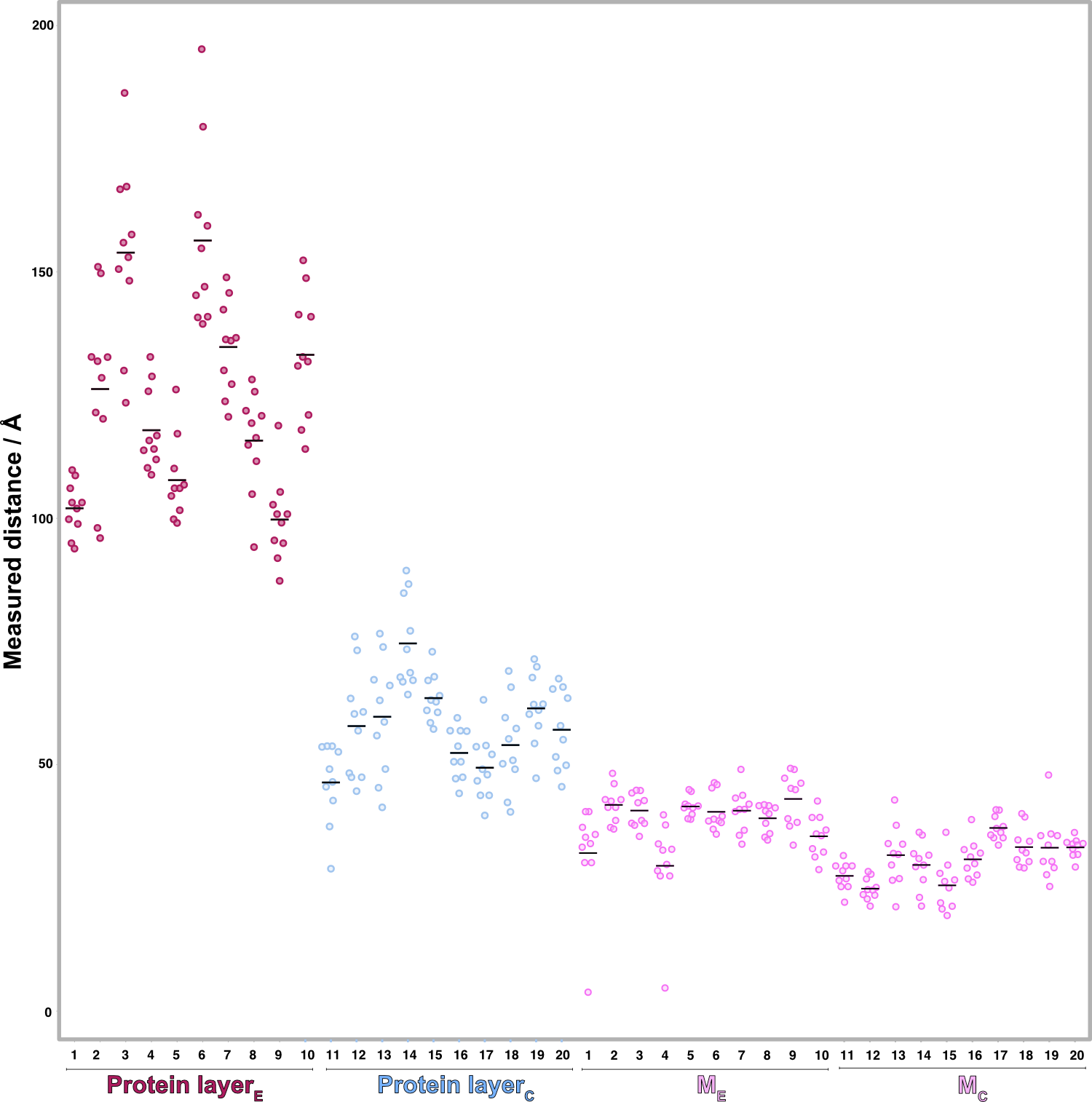

Supplement: S5 Fig — A plot showing the distribution of individual data points measured for the thickness of various features of PTempty and PTcargo tubes (10 tubes each). Thickness was measured on tomographic projections along the length of the tubes and the mean value for each measurement is indicated for each tube. The individual data points were utilized to derive measurements shown in Fig 3. The raw data underlying this figure can be found in the S1 Data. (PNG) [file pbio.3002533.s011.png]
