# Supplementary material for: Ultrastructural insights into the microsporidian infection apparatus reveal the kinetics and morphological transitions of polar tube and cargo during host cell invasion
Source: PLoS Biol. 2024 Feb 29;22(2):e3002533. doi: 10.1371/journal.pbio.3002533 (PMC10931468; doi:10.1371/journal.pbio.3002533)
Supplement: S1 Table — (PDF) [file pbio.3002533.s004.pdf]

S1 Table. Cryo-ET data collection parameters.

Data collection parameters	
Microscope	Titan Krios G2
Voltage (kV)	300
Camera	Gatan K2
Energy filter	Yes, BioQuantum
Magnification	64000x
Total Electron dosage (e–/$Å^2$)	110 to 120
Defocus range (µm)	-1.5 to 5
Tilt range (°)	-60 to +60
Tilt angle increment (°)	2/3
Frames per movie	10
Object pixel size (Å) at super-resolution mode	2.173
Number of tomograms	50
