# Supplementary material for: Ultrastructural insights into the microsporidian infection apparatus reveal the kinetics and morphological transitions of polar tube and cargo during host cell invasion
Source: PLoS Biol. 2024 Feb 29;22(2):e3002533. doi: 10.1371/journal.pbio.3002533 (PMC10931468; doi:10.1371/journal.pbio.3002533)
Supplement: S2 Table — The list of mass spectrometry hits is arranged according to peptide-spectrum match (PSM). The number of consecutive histidines (3xHis, 4xHis, 7xHis) in the protein is listed. PTP3 is the only protein with 7 consecutive histidines. (PDF) [file pbio.3002533.s005.pdf]

S2 Table. Mass spectrometry results of a PTP3 affinity-purified sample. The list of mass spectrometry hits is arranged according to peptide-spectrum match (PSM). The number of consecutive histidines (3xHis, 4xHis, 7xHis) in the protein is listed. PTP3 is the only protein with seven consecutive histidines.

Accession Code	Description	Coverage [%]	# Peptides	# PSMs	# Unique Peptides	MW [kDa]	3xHis	4xHis	7xHis	%Disorder
VNE6901_135	Polar tube protein 3 (PTP3)	71	99	591	99	151	2	1	1	56.40
VNE6904_200	Polar tube protein 2 (PTP2)	55	14	30	14	30.1	0	0	0	15.66
VNE6909_014	Ricin B lectin (RBL7)	47	13	28	13	24.3	0	0	0	7.18
VNE6901_309	Ricin B lectin (RBL3c)	52	13	16	13	25.2	0	0	0	9.29
VNE6901_034	Ricin B lectin (PTP6a)	60	12	200	12	24.3	0	0	0	13.27
VNE6910_036	Ricin B lectin (RBL4b)	62	12	131	12	21.2	0	0	0	17.74
VNE6901_039	Ricin B lectin (PTP6d)	64	11	75	11	23.3	1	1	0	16.42
VNE6909_187	hypothetical SP-containing protein	36	10	17	10	33.3	0	0	0	34.62
VNE6901_358	Lysine-tRNA ligase (KARS1)	19	10	13	10	59.4	0	0	0	5.18
VNE6909_165	Ribosomal protein eS1	38	9	13	9	26.9	0	0	0	13.33
VNE6911_083	Heat shock protein 70-like protein	16	9	9	9	74.5	0	0	0	14.78
VNE6904_210	hypothetical SP-containing protein	62	8	26	8	15.6	0	0	0	3.70
VNE6903_132	Ribosomal protein MDF2	51	8	12	8	24	0	0	0	63.37
VNE6901_289	Triosephosphate isomerase	26	7	9	7	26.9	0	0	0	2.50
VNE6912_035	hypothetical membrane protein	14	7	9	7	48.2	0	0	0	54.42
VNE6903_264	Putative endonuclease/exonuclease/phosphatase family protein	27	7	8	7	33.3	0	0	0	2.34
VNE6905_175	Ribosomal protein uS4	36	7	8	7	21.7	0	0	0	21.35
VNE6907_076	Ribosomal protein eS4	27	7	7	7	30	0	0	0	7.87
VNE6902_049	Ribosomal protein uL24	43	6	9	6	16.1	0	0	0	22.70
VNE6903_258	Ricin B lectin (RBL4c)	31	6	9	6	21.1	0	0	0	6.45
VNE6901_023	Protein argonaute-2 (AGO2)	8	6	6	6	92.2	0	0	0	10.92
VNE6903_024	Putative splicing factor	18	5	12	5	37.3	0	0	0	36.14
VNE6901_040	Ricin B lectin (RBL4d)	25	5	9	5	21.7	0	0	0	25.93
VNE6906_226	Ribosomal protein uL2	21	5	7	5	26	0	0	0	22.92
VNE6905_120	Retrovirus-related Pol polyprotein from transposon Pr125Pol	4	5	5	5	136.7	0	0	0	11.01
VNE6911_031	hypothetical protein	27	4	7	4	13.3	0	0	0	96.03
VNE6901_014	hypothetical protein	34	4	5	4	19.4	0	0	0	23.81
VNE6907_050	Ribosomal protein uL14	37	4	5	4	16	0	0	0	15.54
VNE6912_194	Ribosomal protein uL6	22	4	5	4	21	0	0	0	2.65
VNE6902_098	Ribosomal protein eL19	23	4	4	4	18.6	0	0	0	18.52
VNE6907_057	Ribosomal protein uS15	29	4	4	4	17	0	0	0	12.58
VNE6911_140	Ribosomal protein eL32	20	3	5	3	16.1	0	0	0	13.48
VNE6903_263	Ribosomal protein eL24	29	3	4	3	10.3	0	0	0	22.83
VNE6903_368	Ricin B lectin (RBL8)	19	3	4	3	21	0	0	0	11.96
VNE6911_060	Ribosomal protein uS9	18	3	4	3	17.5	0	0	0	14.01
VNE6901_104	Nuclear segregation protein BFR1-like	9	3	3	3	45.6	0	0	0	19.80
VNE6902_200	Putative subtilisin-like proteinase	5	3	3	3	51	0	0	0	13.67
VNE6912_127	Elongation factor 1-alpha 1 (EF1A1)	10	3	3	3	51.1	0	0	0	7.43
VNE6901_035	Ricin B lectin (PTP6b)	14	2	2	2	21.4	0	0	0	8.56
VNE6901_308	Ricin B lectin (RBL3b)	10	2	2	2	20.3	0	0	0	3.35
VNE6901_368	Ribosomal protein eL27	13	2	2	2	14.5	0	0	0	2.34
VNE6902_064	High mobility group protein	8	2	2	2	24	0	0	0	43.81
VNE6902_107	Ribosomal protein uL18	7	2	2	2	34.4	0	0	0	15.05
VNE6904_059	Integrase catalytic domain-containing protein	4	2	2	2	70.3	0	0	0	70.34
VNE6908_017	Histone H2A (H2A)	15	2	2	2	13	0	0	0	24.39
VNE6910_078	Ribosomal protein uS11	16	2	2	2	14.4	0	0	0	19.71
VNE6904_195	Polar tube protein 1 (PTP1)	5	1	2	1	41.6	0	0	0	46.93
VNE6905_241	Nuclear transcription factor Y subunit (nfya-1)	9	1	2	1	14.6	0	0	0	49.19
VNE6910_006	hypothetical protein	1	1	2	1	66.5	0	0	0	16.32
VNE6910_107	Ribosomal protein eL34	10	1	2	1	12.3	0	0	0	12.15
VNE6901_044	Ribosomal protein eS24	16	1	1	1	15.5	0	0	0	34.07
VNE6901_069	Aldose reductase	2	1	1	1	34.8	0	0	0	1.30
VNE6901_293	Ribosomal protein eS21	22	1	1	1	7.6	0	0	0	8.82
VNE6901_343	Ribosomal protein uS2	4	1	1	1	29.6	0	0	0	18.28
VNE6901_372	Histone domain-containing protein	5	1	1	1	16.4	0	0	0	31.29
VNE6901_379	Ribosomal protein uS17	9	1	1	1	18.2	0	0	0	11.88
VNE6902_019	hypothetical SP-containing protein	6	1	1	1	21	0	0	0	22.45
VNE6902_041	Ribosomal protein uS19	6	1	1	1	16	0	0	0	18.62
VNE6902_092	Ribosomal protein uS13	9	1	1	1	18	0	0	0	16.05
VNE6902_093	Ribosomal protein msL1	11	1	1	1	8.8	0	0	0	32.43
VNE6903_164	hypothetical protein	2	1	1	1	45.8	0	0	0	12.85
VNE6904_037	Ribosomal protein uS5	4	1	1	1	25.9	0	0	0	8.33
VNE6904_069	Serpin-type proteinase inhibitor 2	2	1	1	1	40.6	0	0	0	1.97
VNE6904_088	Ribosomal protein eL40	7	1	1	1	14.2	0	0	0	17.05
VNE6904_207	uncharacterized protein	4	1	1	1	21.8	0	0	0	6.99
VNE6905_023	Ribosomal protein eS26	13	1	1	1	12.1	0	0	0	27.62
VNE6905_101	Ribosomal protein eS6	8	1	1	1	24.9	0	0	0	15.00
VNE6905_111	Zinc finger C2H2 domain-containing protein	3	1	1	1	32.9	0	0	0	30.50
VNE6905_169	hypothetical SP-containing protein	3	1	1	1	30.5	0	0	0	8.65
VNE6905_231	Spore wall protein 8-like protein	7	1	1	1	12.4	0	0	0	18.52
VNE6907_026	Glutaredoxin	8	1	1	1	15.7	0	0	0	2.22
VNE6907_113	Ribosomal protein eL20	6	1	1	1	20.9	0	0	0	9.89
VNE6907_188	uncharacterized protein	8	1	1	1	11.8	0	0	0	49.07
VNE6908_041	hypothetical protein	4	1	1	1	21.8	0	0	0	9.52
VNE6908_165	Ribosomal protein eS28	16	1	1	1	7.6	0	0	0	23.53
VNE6908_188	Eukaryotic translation initiation factor 5A	5	1	1	1	17.8	0	0	0	17.83
VNE6908_192	Putative glutaredoxin	12	1	1	1	10.2	0	0	0	13.79
VNE6909_015	Ribosomal protein eL43	10	1	1	1	9.8	0	0	0	13.33
VNE6910_008	Ribosomal protein uS8	9	1	1	1	14.5	0	0	0	0.78
VNE6910_066	hypothetical SP-containing protein	2	1	1	1	43.9	0	0	0	75.66
VNE6910_158	Ribosomal protein uL3	2	1	1	1	43.1	0	0	0	17.44
VNE6911_029	Ribosomal protein uL29	9	1	1	1	14.8	0	0	0	12.90
VNE6912_041	Ribosomal protein eL30	11	1	1	1	11.8	0	0	0	14.68
VNE6912_114	hypothetical SP-containing protein	4	1	1	1	26.3	0	0	0	38.46
