# Supplementary material for: Ultrastructural insights into the microsporidian infection apparatus reveal the kinetics and morphological transitions of polar tube and cargo during host cell invasion
Source: PLoS Biol. 2024 Feb 29;22(2):e3002533. doi: 10.1371/journal.pbio.3002533 (PMC10931468; doi:10.1371/journal.pbio.3002533)
Supplement: S3 Table — The 2 prominent protein bands indicated by an asterisk (*) in Fig 4C, one around 130 kDa and 20 kDa were sliced and analyzed. The list of mass spectrometry hits is arranged according to peptide-spectrum match (PSM). (PDF) [file pbio.3002533.s006.pdf]

S3 Table. In-gel mass spectrometry analysis of PTP3 affinity-purified sample. The two prominent protein bands indicated by an asterisk (*) in Figure 4c, one around kDa and 20 kDa were sliced and analyzed. The list of mass spectrometry hits is arranged according to peptide-spectrum match (PSM).

Description	Coverage [%]	# Peptides	# PSMs	# Unique Peptides	MW [kDa]	# Peptides: (~150 kDa)	# Peptides (~20 kDa)
PTP3	89	153	2271	153	151.00	150	33
uncharacterized protein	12	10	20	10	91.60	10	
DNA-directed RNA polymerase I subunit RPA190 (RPA1)	9	10	15	10	157.40	10	
elongation factor 1-alpha 1 (EF1A1)	26	7	9	7	51.10	4	1
DNA-directed RNA polymerase II subunit RPB2	55	58	94	58	131.70	3	
hypothetical protein	2	1	6	1	84.80	1	
ricin B lectin (PTP6a)	80	17	246	17	24.30	2	17
polar tube protein 1 (PTP1)	9	2	4	2	41.60	1	
ribosomal protein eL13	23	3	5	3	18.90		3
hypothetical membrane protein	17	8	10	8	48.20		8
ricin B lectin (PTP6c)	11	3	6	1	20.20		3
ribosomal protein uL13	17	3	3	3	22.30		3
uncharacterized membrane protein	29	4	7	4	21.50		4
ricin B lectin (RBL4c)	35	3	4	3	21.70		3
ricin B lectin (RBL4a)	31	4	6	4	21.20		4
ribosomal protein eL19	18	3	4	3	18.60		3
proteasome subunit PSB4 (PSB4)	7	1	1	1	22.00		1
polar tube protein 2 (PTP2)	30	8	16	8	30.10		8
ricin B lectin (RBL3c)	9	2	2	2	25.20		2
ricin B lectin (PTP6d)	72	16	80	14	23.30		16
putative splicing factor	22	6	7	6	37.30		6
spore wall protein 25-like	12	2	2	2	25.80		2
RNA-binding protein	8	1	1	1	20.60		1
ribosomal protein uL2	8	1	1	1	26.00		1
ribosomal protein uS7	5	1	1	1	20.90		1
retrovirus-related pol polyprotein from transposon Pr125pol	6	7	10	7	136.70		7
eukaryotic translation initiation factor 5A	43	8	14	8	17.80		8
proteasome subunit PSB3 (PSB3)	16	4	4	4	23.00		4
60S ribosomal protein L20	59	11	15	11	20.90		11
ricin B lectin (PTP6b)	47	8	14	8	21.40		8
ubiquitin-like protein	4	1	2	1	36.10		1
hypothetical SP-containing protein	34	4	4	4	19.40		4
DNA-directed RNA polymerase III subunit (RPC2)	8	7	7	7	123.90		
hypothetical SP-containing protein	13	2	2	2	21.00		2
hypothetical SP-containing membrane protein	7	1	1	1	21.10		1
glutaredoxin-3 (GRX3)	32	5	8	5	22.10		5
hypothetical SP-containing protein	8	1	1	1	33.30		1
spore wall protein 26	9	2	2	2	24.70		2
proteasome subunit PSB5 (PSB5)	31	4	4	4	25.20		4
hypothetical SP-containing protein	9	1	1	1	12.40		1
separin (ESP1)	2	1	1	1	92.10		1
zinc finger C2H2 domain-containing protein	19	4	4	4	32.90		4
triosephosphate isomerase	11	2	2	2	26.90		2
integrase catalytic domain-containing protein	4	2	2	2	70.30		2
proteasome subunit PSB1 (PSB1)	14	3	3	3	23.50		3
uncharacterized protein	7.00	1.00	5.00	1.00	24.50		1
