# Supplementary material for: Ultrastructural insights into the microsporidian infection apparatus reveal the kinetics and morphological transitions of polar tube and cargo during host cell invasion
Source: PLoS Biol. 2024 Feb 29;22(2):e3002533. doi: 10.1371/journal.pbio.3002533 (PMC10931468; doi:10.1371/journal.pbio.3002533)
Supplement: Attachment — Submitted filename: 137142_1_rebuttal_2820840_s2hb8y.pdf [file pbio.3002533.s012.pdf]

Full Revision

Manuscript number: RC- 2023-02037
Corresponding author(s): Himanshu, Sharma, Jonas, Barandun

1. General Statements

We thank the three reviewers for reading our manuscript and providing constructive input. Below we have addressed the comments point by point and believe that the manuscript has benefited from the suggested changes.

In addition to the requests made by the reviewers, we have also added new data on the mass spectrometry analysis of individual protein bands from the SDS PAGE gel shown in Figure 3, which allowed us to provide more information identifying PTP3-associated factors.

2. Point-by-point description of the revisions

Reviewer #1 (Evidence, reproducibility and clarity (Required)):

Summary: Sharma, et al. report the characterization of the polar tube (PT) from the microsporidian species, Vairimorpha necatrix, using a combination of optical microscopy, cryo-ET, and proteomics. The polar tube is a fascinating invasion apparatus which mediates the translocation of the parasite into the inside of a host cell to initiate infection. Similar to results obtained previously in other species, the authors show that PT firing in Vairimorpha necatrix is extremely fast, occurring on the order of 1 sec, and that the extruded PT is over 100 microns long in this species. Using cryo-ET to image the PT at a high resolution, they find that it exists in two major states: both an empty state and a state filled with cargo, and that the thickness of the tube wall changes when cargo is present. Strikingly, the authors observed that one of the cargo components, the ribosomes, are organized ordered array that may have helical symmetry. Finally, the authors took advantage of a naturally occurring "His tag" on PTP3 to affinity purify PTP3-containing protein complexes and analyze the composition using proteomics.

We thank reviewer #1 for the positive comments.

MAJOR COMMENTS:

1. ln 139-140: The absolute handedness of something can be very tricky to determine by cryo-ET (but certainly is possible). Variable hardware configurations between microscopes and differing conventions between software packages (e.g., for what direction is a positive tilt angle) can lead to inversion of the apparent handedness in the final tomogram. How certain are the authors that the absolute handedness is indeed right handed, as this seems to vary between the various display items in the manuscript? For example, in Fig 1c, my impression is that ribosome helices are left handed, as they are also in the supplemental movie. If this isn't known with certainty, perhaps it would be sufficient to describe the apparent helical symmetry but state that the handedness is

ambiguous.

We have attempted to determine the absolute handedness by visual inspection of the reconstructed tomograms and the sub-tomogram average of the ribosomes. In the absence of complementary confirmatory information and with the challenges and uncertainties associated with determining the absolute handedness of spirals by cryo-ET, we agree that it is more appropriate to leave the handedness ambiguous. Since the handedness is not critical for our findings, we have removed the handedness and refer to the spirals as helical throughout the text, e.g.:

> *"The particles were arranged in an array-like pattern (**Fig. 2a**), wherein their parallel alignment formed a ~~right-handed~~ helix traversing the entire length of the reconstruction (**Fig. 2b**)."*

MINOR COMMENTS:

2. ln 39-40: Perhaps also cite the E. cuniculi genome paper?

We have added the following suggested reference to the section about genome compaction:

> *Katinka MD, Duprat S, Cornillott E, Méténler G, Thomarat F, Prensier G, Barbe V, Peyretaillade E, Brottier P, Wincker P, et al (2001) Genome sequence and gene compaction of the eukaryote parasite Encephalitozoon cuniculi. Nature 414: 450–453*

3. ln 97-98: It is interesting that the PT shortens in V. necatrix as well, and while I can pick this out in some of the individual traces in Sup Fig. 1b, it seems to get washed out in the trend line and isn't super obvious. If it isn't to laborious, it could be nice to add a panel showing the quantification of this (e.g., plotting the final length of each PT as a percentage of the maximum length achieved).

The retraction in polar tube length is indeed visible in the individual traces, and we have, as suggested by the reviewer, updated **Supplementary Figure 1C** to plot the final length next to the maximal length depicting PT shortening. The updated Figure (below) now shows in addition to the maximal length, the length at the end of germination. Further, and in response to reviewer #2 point 8, individual data points are depicted in all plots in panel C. Also, S and SP are now defined in the figure legend as a response to reviewer #1 point 15.

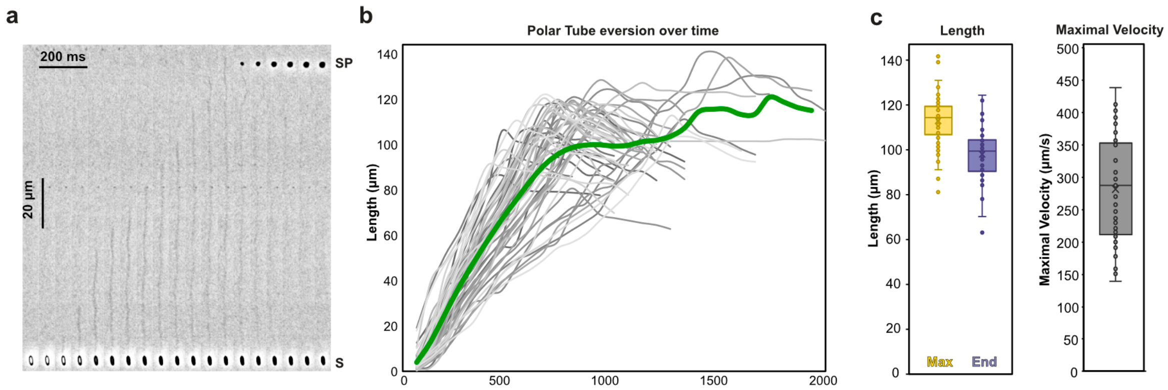

*"**Supplementary Figure 1. Tracking polar tube eversion to understand germination dynamics and tube length.** (**a**) A kymograph obtained via live light microscopy analysis of polar tube firing events from Vairimorpha necatrix. **The spore at the bottom of the kymograph is denoted by 'S' and the sporoplasm ejected on the distal end is indicated as 'SP'.** (**b**) Length over time diagrams of all analyzed polar tube eversion events. The average length over time is colored in green. (**c**) Bar plots of polar tube maximal length (yellow), **length at the end (blue)**, and maximum velocity distribution (grey)."*

4. Ln 98-100: Strictly speaking, I don't think the referenced figure shows the sporoplasm being transformed into an extended conformation, only that it is spherical upon exit. Simply reword this to make clear that the deformations are inferred to occur but not directly observed.

The reviewer is correct about the figure not showing an extended state in the polar tube during translocation. However, such a transformation must occur as the sporoplasm is pushed through the narrow tube, and this has recently also been shown very nicely by Ray Chang et al, 2023. As suggested, we have reworded the section about sporoplasm transformation upon exit and state now that the sporoplasm *likely* transforms from restricted to extended and to spherical conformation upon exit. In addition, we added the reference to Ray Chang *et al.*, 2023 eLife. https://doi.org/10.7554/eLife.86638.1). The section, in line 87ff, reads now:

*"Akin to tube remodeling events, the sporoplasm **also likely transforms from a restricted** spore state to extended conformation during extrusion **[30]**, into a **spherical** shape upon emergence (**Supplementary Fig. 1a**), and these events may impose immediate reorganization of subcellular structures."*

5. Because PT firing is so fast, the probability of trapping a PT in the process of transporting cargo would be pretty low. So then why does the PT still contain cellular cargo like ribosomes inside in the tomograms? Should these not have emerged in the sporoplasm which would enter the host cell? Are these "defective" spores that have failed to complete sporoplasm transport? Perhaps this is worth discussing.

The reviewer is right: once initiated, PT firing in solution is fast. However, we see distinct structural states (PTcargo and PTempty) in our tomograms. This clearly shows that not all PTs were in the same, final post-ejection state upon cryo-ET imaging. We can only speculate on the

reasons for this. Based on how we prepare the grids, it is likely that spores are still in the process of germination when we expose the mixture to the EM grid. It is possible that the interaction with the grid support traps some PTs in a non-final state with sporoplasms inside the tube. We think that this may happen e.g., by the grid reducing the flexibility of the PT which might be needed to pump and release the SP – i.e., preventing the retraction phase.

As this is speculative, we have added the following new section in the discussion (lines 218ff) to highlight these additional aspects to be kept in mind while interpreting our findings:

> ***"Here, we probe cargo delivery by cryogenically preserving snapshots of the ultrarapid spore germination process. Congruence in polar tube firing kinetics seen in our work and previous studies [23,25], suggest a conserved theme of sporoplasm movement upon germination (Supplementary Fig. 1) between different microsporidian species. Firing does not occur simultaneously across all spores, and the implementation of on-grid germination successfully captured heterogeneous states of the PT and sporoplasm by immediately vitrifying spores upon inducing firing. It is possible that the mechanical interaction of the PT with the grid support limited tube flexibility, thus slowing down the rapid firing, and trapped otherwise transient states. Two caveats are worth mentioning in relation to this analysis. Firstly, any ordering of the observed structural states onto a timeline is only hypothetical, since cryo-ET does not provide any time resolution. Secondly, we cannot completely rule out that some tomograms represent misfired tubes that fail to fully deliver cargo [25]. However, we made deliberate efforts to avoid imaging such PTs by actively excluding tubes that exhibited bloated appearances and extremely high electron density during the data collection process."***

6. ln 118: The authors note an apparent correlation between the phase of germination and the thickness of the tube wall but don't specify what this correlation is. Is it thicker in the early phase and thinner in later phase, or vice versa? One could imagine "empty" tubes existing before or after sporoplasm transport, for example, so I'm not sure I follow how the phase is being inferred from the tomograms.

This observation is correct, and we are now highlighting this in the main text. As we cannot with high confidence distinguish pre- from post-germination polar tubes, we have added a section describing that the observed polar tubes could represent either of the two states, on line 109ff:

> ***"Here, it is worthwhile to note that the PTcargo or PTempty states represent static snapshots after firing and could represent states before or after sporoplasm translocation."***

See also the updated discussion section in response to the related comment (no. 5), lines 218ff.

It is also worth mentioning that independent of the temporal state of the PT during the germination phase, the elongated tube lacking cargo has a reduced diameter but thicker outer wall, which suggests a certain intrinsic flexibility and ability for major ultrastructural remodeling.

7. ln 119-120: What is the evidence that the outer layer is made of PTPs, or that it is even protein (for example, as opposed to cell wall-like carbohydrate polymers)? I think this seems like a very reasonable hypothesis, but I would suggest explaining the logic and ensuring the degree of uncertainty is conveyed clearly. In light of this, I would also suggest changing figure labels, etc, that refer to the PTP layer (e.g., Fig. 3, PTPc and PTPe labels).

As described in lines 157ff:

> *"The presence of an outer protein-composed tube wall also agrees well with immunolabeling studies where fluorescently labeled antibodies against individual PTPs localized to the outer sheath of the tube wall (Han et al, 2017)."*

To strengthen this further, we have added earlier in the main text (line 113ff) additional references to previous studies showing an outer proteinaceous layer surrounds the tube:

> *"… flanked by an outer layer of polar tube proteins (light blue and magenta arrows,* ***Fig. 1 & Supplementary Fig. 2****).* ***These observations are also in line with previous reports of an outer proteinaceous layer in germinated PTs [29,36,37]."***

29. Takvorian PM, Han B, Cali A, Rice WJ, Gunther L, Macaluso F, et al. An Ultrastructural Study of the Extruded Polar Tube of Anncaliia algerae (Microsporidia). J Eukaryot Microbiol. 2020;67: 28–44. doi:10.1111/jeu.12751

36. Xu Y, Takvorian PM, Cali A, Orr G, Weiss LM. Glycosylation of the major polar tube protein of Encephalitozoon hellem, a microsporidian parasite that infects humans. Infect Immun. 2004;72: 6341–6350. doi:10.1128/IAI.72.11.6341-6350.2004

37. Fayet M, Prybylski N, Collin M-L, Peyretaillade E, Wawrzyniak I, Belkorchia A, et al. Identification and localization of polar tube proteins in the extruded polar tube of the microsporidian Anncaliia algerae. Sci Rep. 2023;13: 8773. doi:10.1038/s41598-023-35511-y

In addition, we now changed the PTP labels to "PT layer" for the "Polar Tube layer" in the figures.

8. ln 121, 123: "sheathed by a thin layer" and "enveloped by a thick outer layer": is this an additional layer being described? Or is this referring to the putative PTP layer, and that its thickness is variable?

This refers to the same outer putative PTP layer. We have aligned both naming instances to "enveloped" and hope this makes it more consistent (lines 106ff):

> *"Polar tubes filled with dense cellular cargo (PTcargo) (****Fig. 1a-c****) had diameters of more than 120 nm and are* ***enveloped by a thin layer****; in contrast, the electron-lucent or empty tubes (****Fig. 1d, e****) have a diameter of significantly less than 120 nm but were* ***enveloped by a thick outer layer*** *(PTempty) (****Supplementary Fig. 2b****)."*

ln 125-126: While I understand how some features, like ribosomes, proteasomes, and generic membrane compartments could be identified, it is unclear to me how one would recognize the

Review COMMONS

nucleus when inside the PT, nor are any examples shown. If the data is clear, perhaps the authors could show it in a figure? Otherwise, I suggest removing the claim regarding the nucleus.

We agree and have removed the claim in lines 115-117:

> *"Most interestingly, across all our tomograms, we observed different internal cargo, including electron-dense material inside membranous compartments ~~**that could be attributed to organelles like the nucleus**~~, randomly oriented or highly ordered large molecular complexes such as ribosomes and **proteasome-like particles**, and empty vesicles or empty tubes."*

9. The arrangement of the ribosomes in a subset of tubes is really fascinating! While the number of observations is relatively small (n=5), it seems like it should be possible to comment preliminarily on whether there is much variability in their helical arrangement. Do the helical parameters vary much between observations? Does the til, pitch, etc vary much, are the 5 occurrences very similar? Is there any sign that they are associated with a membrane? Also, since the ribosomes form a lattice-like arrangement, it seems like it would be possible to trace ribosome helices in both the left and right handed directions. How did the authors decide between the two possibilities? This doesn't seem to be discussed.

We agree that the discovery of this arrangement was very unexpected and fascinating. We have now complemented **Supplementary Figure 3** with the segmented reconstructions of additional regions. While it is apparent in both additionally depicted segmentations that ribosome arrays cluster close to the outer layer, the spiral arrangement appears to differ between the individual reconstructions. We assume that there is some heterogeneity that might also be induced by the difference in the curvature of the tube. Therefore, parameters such as pitch or tilt most likely vary between the individual occurrences. As mentioned above, we have removed all handedness claims throughout the entire manuscript.
The updated **Supplementary Figure 3** is shown below. and as a response to comment nr. 16, we updated the orientation of the schematic PT coils in panel A based on the current literature.

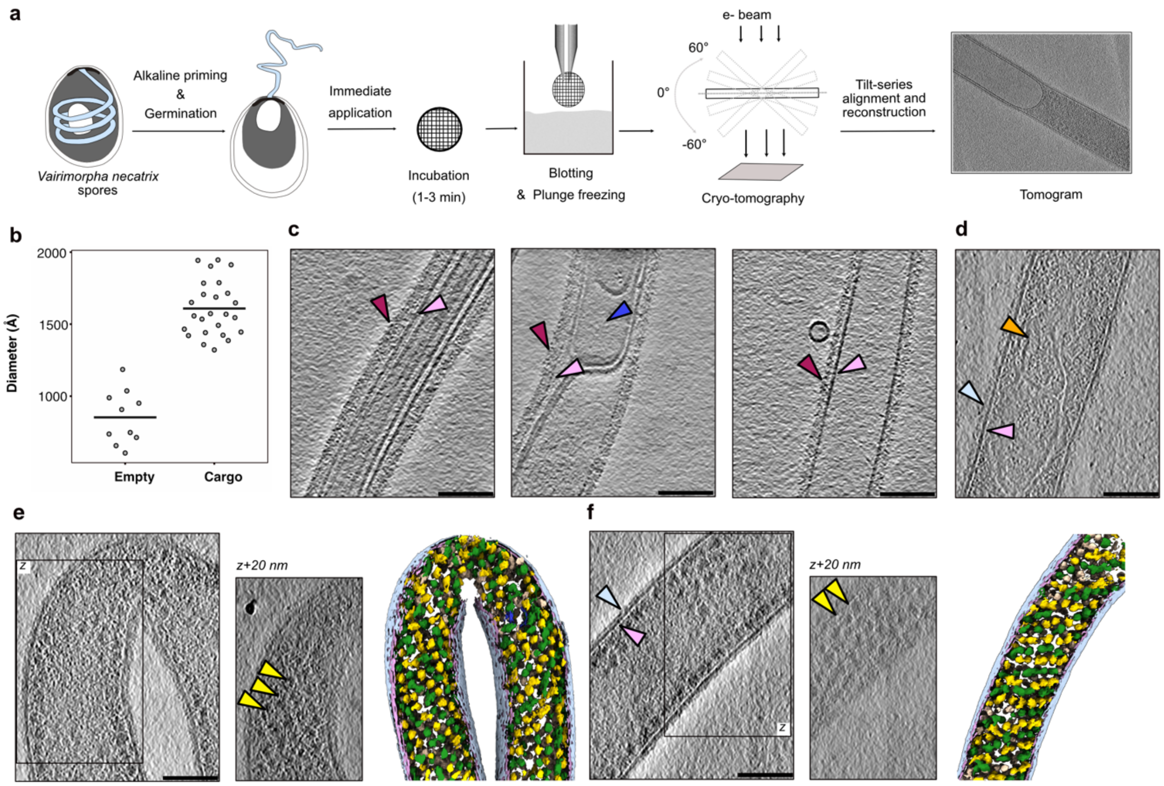

*“**Supplementary Figure 2. Representative tomograms depicting empty or sporoplasm-packed germinated polar tubes.** (**a**) A schematic representation of the methodology for on-grid freezing and collecting tomograms of germinated polar tubes. (**b**) A graph representing the internal diameter of polar tubes (PTempty & PTcargo) visualized using cryo-ET. Each dot represents one tube, and the line represents the mean diameter. (**c**) Representative tomograms of PTempty, or polar tubes devoid of cellular cargo or filled with electron-lucent material. The central section of a tomogram is shown with regions of interest indicated with arrows (magenta for the outer wall, pink for the lipid bilayer, and blue for vesicles). (**d-f**) Representative tomograms from PTcargo, or polar tubes filled with cellular cargo where (e-f) contained ribosome spirals inside tubes. The central section of a tomogram is presented, and regions of interest are indicated with arrows (light blue for the outer tube wall, pink for the lipid bilayer, and yellow for ribosomes). For (e-f), additional views corresponding to the boxed regions, **and corresponding segmented tomograms** are also presented. “*

10. Fig. 2e: Are the two different colors/orientations meant to represent the two protamers of the ribosome dimer? When refined subvolumes are mapped back onto the original tomogram do the authors observe a similar crystalline arrangement of particles as in their segmentation? Are the orientations of the ribosomes correlated, and do the provide any evidence for the dimeric arrangement mentioned? The PlaceObjects plugin for Chimera can be very helpful for visualizing this: https://www.biochem.mpg.de/7939908/Place-Object

The different colors should serve as a visual guide for the reader and show the spiral arrangement of the ribosomes along the tube more clearly.

In response to comments from reviewer #3, we have now also obtained a low-resolution map of the ribosome dimer and mapped these sub-volumes back onto the tomogram. This results in a similar crystalline arrangement as shown in the segmentation. We have included the dimer STA and the placement in **Supplementary Figure 4e**:

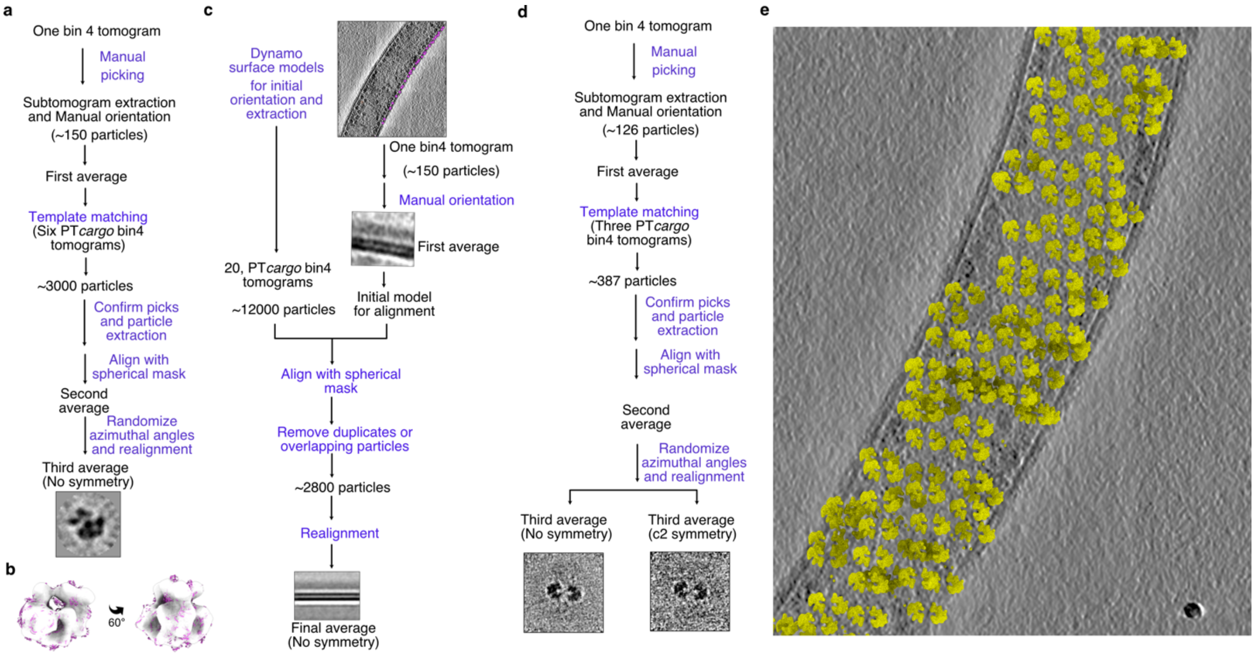

11. Supp figure 4(b-d): Perhaps these models could be colored by pLDDT scores (with a key indicating the color scheme), so the reader can assess the quality of the predictions?

We have added the pLDDT score plots of the top-ranked prediction from the Alphafold output on top of the panel and indicated N and C termini such that it becomes clear that predominantly the C-terminal ends have a lower prediction quality. See updated panel b-d below:

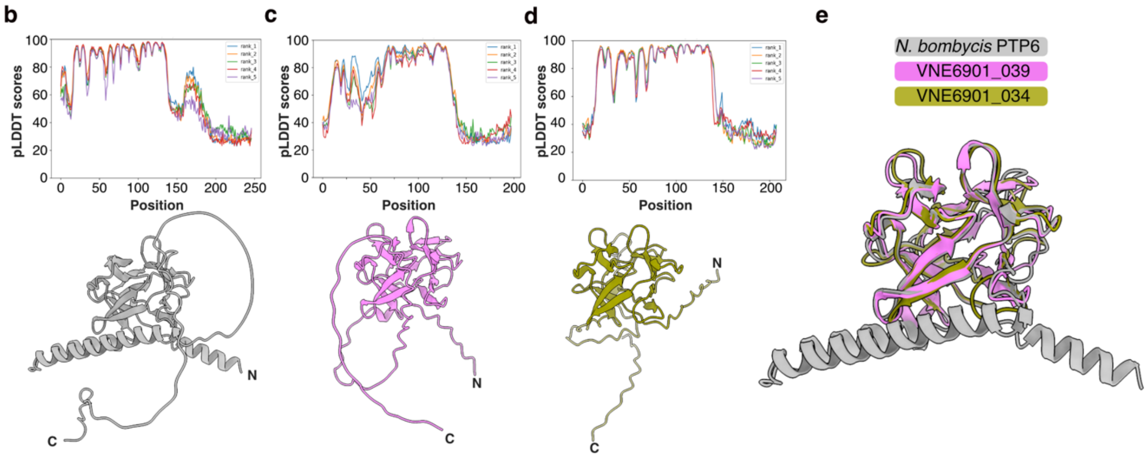

12. How were the measurements of the membrane thickness and putative PTP layer carried out? On the tomogram projections? STAs? How were the boundaries of the layers established (e.g., map threshholding if STA?)? This information appears to be missing from the methods.

We have updated the method section "*Tilt series processing, tomogram generation, segmentation, and* ***distance measurements***" to include more information on how the thickness was measured (lines 330ff):

> ***"The binned, denoised tomogram projections were also used to manually measure membrane thickness and inter-ribosome distances using IMOD and Dynamo. For measurements on PT outer layers, 10 tomograms each from PTempty and PTcargo were surveyed uniformly across their length (n = 10, for each tomogram), to calculate the mean thickness for each tomogram representing each measurement (Figure 3c). "***

We also present the individual measurements for each layer in a new **Supplementary Figure 5** (below).

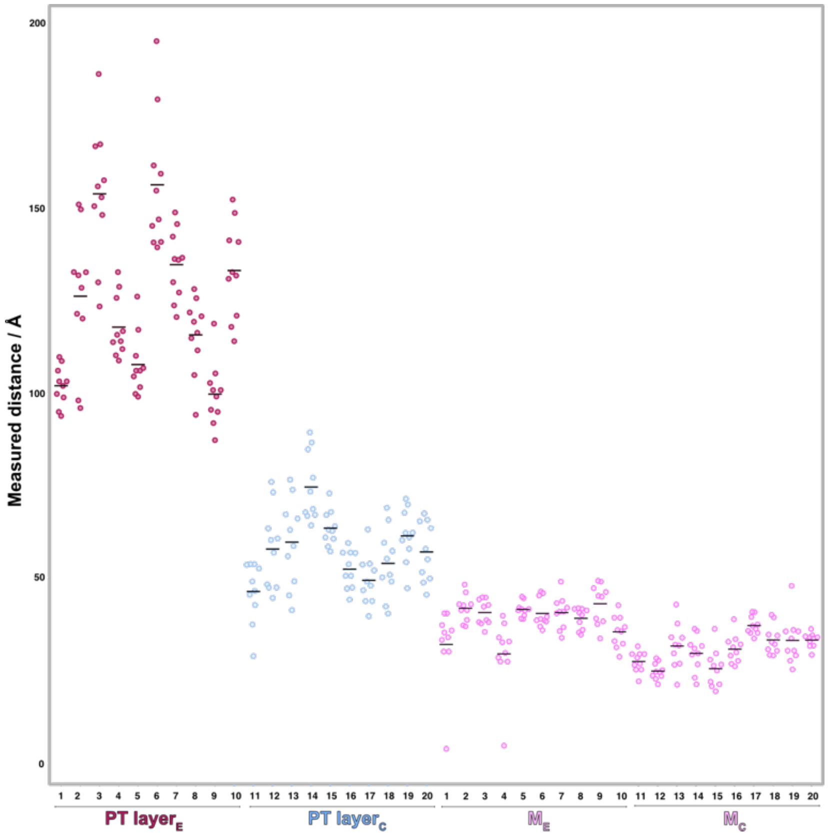

> ***"Supplementary Figure 5: Individual data points for PTP Layer measurements:*** *A plot showing the distribution of individual data points measured for the thickness of various features of PTempty and PTcargo tubes (10 tubes each). Thickness was measured on tomographic projections along the length of the tubes and mean value for each measurement is indicated for each tube. The individual data points were utilized to derive measurements shown in Figure 3."*

13. Some tubes that are labeled as 'PTempty' actually contain cargo and look dense (example supp. Fig 2c, left and middle panels). Is it fair to classify these as empty tubes?

Since the two tube states show clear differences in appearance (outer wall thickness, diameter, internal electron density), and one state has unambiguously cargo inside, we believe that it is fair to label and categorize the second state as empty. We mention in the text that some tubes categorized as empty contain membranous remnants:

> *"Notably, macromolecular complexes were completely missing from PTempty, which mostly housed empty-looking vesicles (dark blue arrows,* ***Fig 1d*** *&* ***Supplementary Fig. 2c****) or no vesicles (****Fig 1e*** *&* ***Supplementary Fig. 2c****)."*

14. Fig. 3d: I am not entirely clear on what is being shown here. Are independent reconstructions of PTcargo and PTempty superposed (aligned on membrane)? The description in the figure legend doesn't clearly say what is being displayed. I think it might be more clear to show these side-by-side instead of superposed (i.e., 4 panels instead of 2).

The initial version of Fig. 3d showed a superposition of the two volumes, segmented, and colored by the outer layer and membrane. The thicker outer layer was shown transparently.

We have now, based on the suggestion from reviewer #1, replaced the panel with side-by-side views of the two segmented and colored reconstructions and adjusted the figure legend to clarify what is being shown. In addition, and as a response to the minor comment from reviewer 3, point 10, we have removed the diagonal lines in the schematic in panel 3e. Further, the PTP labels have now been replaced by "PT-layer".

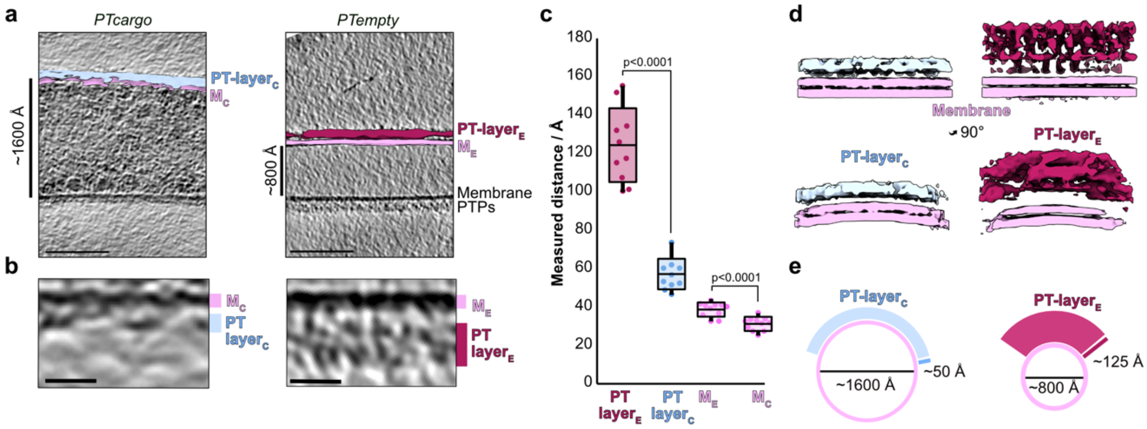

***Figure 3. Visualizing polar tube wall features from cargo-filled and empty tubes.** (a) Central slices through representative cryo-tomograms from cargo-filled and empty polar tubes. Segmentations of the inner and outer layers are superimposed onto one side of the tube in both tomogram slices. The scale bar is 100 nm. (**b**) Zoomed in sections of the tube wall from (a). The scale bar is 10 nm. (**c**) A plot depicting the thickness of each PT layer and membrane bilayer, as measured across various tomograms, for cargo-filled and empty polar tubes. The p-values of two-tailed, unpaired Student's t-test analyses are shown above the compared plots. (**d**) **Side-by-side comparison of two slab views of subtomogram averages obtained from cargo-filled (left) or empty (right) polar tube walls. The volumes were segmented and colored by membrane and outer layer. The views at the top (slabs along the polar tube) are shown below rotated 90° around the Y-axis (slabs across the polar tube).** Map regions are colored as in (a). (**e**) A schematic representation of polar tube and PT layer remodeling during cargo movement.*

Review COMMONS

15. Sup Fig 1: Define S and SP in legend or just spell out on figure? Missing x-axis label on panel b.

We have defined S and SP in the figure legend. The X-axis in panel b was accidentally removed by cropping too generously, which has been corrected in the revised version. See the updated version of the figure under Reviewer # 1 – comment 3.

16. Fig. 4b and Sup Fig 2a: The depictions of the PT in the spore here are left-handed. In a few species, the coil of the PT was found to form a right-handed helix (Jaroenlak, et al.), and it seems plausible that this may be a general feature that would be conserved across microsporidia. I appreciate that it might not be actually known to be right-handed in V. necatrix, but if there is no strong data either way, perhaps it would make sense for these depictions of the PT to be right-handed.

We thank reviewer #1 for very carefully checking the figures and spotting this mistake. The depictions of the coils have been adjusted in **Fig. 4b** and **Supplementary Figure 2a** (see also response to reviewer #1 - point 9).

Fig 4 panel b with corrected coil orientation in the schematic spore:

b

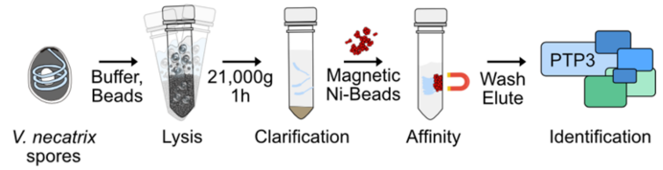

I think all three of us are more or less in consensus about this manuscript, and I largely agree with the other reviewers comments. I think after addressing reviewer suggestions, this will be a pretty nice story.

Thank you once again for the careful assessment, the very productive review, and the constructive comments. We are very glad to hear that our work resulted in a pretty nice story and hope that we addressed all concerns in the revised version.

Reviewer #1 (Significance (Required)):

Overall, this manuscript from Sharma, et al. presents interesting new findings about the structure and cargo transport function of the microsporidian PT. Microsporidia infect a wide range of hosts, including humans, and how the PT mediates parasite entry into cells is poorly understood. The approaches used in this study are appropriate for tackling the questions at hand, and appear to be generally well executed and interpreted. The observation that ribosomes assemble into an array within the PT is very unexpected and quite fascinating, and may be of broader interest to researchers working on ribosome structure and function, in addition to researchers studying

microsporidia. The approach to investigating proteins interacting with PTP3 was quite elegant, and yielded a list of potential interactors that appears to be of very high quality and is highly plausible based on the literature field. We think this work is a substantial advance in the field and provides important new insights into the organization of the PT.

- Please define your field of expertise with a few keywords to help the authors contextualize your point of view:
Structural biology, microsporidia

Indicate if there are any parts of the paper that you do not have sufficient expertise to evaluate.
-We are not experts in proteomics/mass spectrometry

Reviewer #2 (Evidence, reproducibility and clarity (Required)):

In this paper Sharma et al. use cryo-electron tomography to study structural properties of the polar tube invasion apparatus from the microsporidian parasite Vairimorpha necatrix. The main conclusions of the paper are related to the unique organization of ribosomes in the polar tube, and the organization of the surface layer of the tube. The cryo-ET data presented in this paper are of high quality, and add new insights into the structure of the polar tube, which have not been reported previously. The authors also purify an endogenous polar tube protein, PTP3, via a native cluster of histidines, and identify co-purifying proteins, which provides new insights into the proteins present in the polar tube that may interact directly or indirectly with PTP3. The endogenous purification was innovative and well carried out, a very nice result.

We thank reviewer #2 for the positive assessment of our study and are glad to hear that the cryo-ET data are considered of high quality. Thank you also very much for your nice comment about the endogenous purification strategy.

Major comments:

1) Our biggest comment on this manuscript is that we feel the cryo-ET data are often over-interpreted. We would like to request the authors to ensure that their conclusions are justified by the data. We realize that this is a general weakness of cryo-ET at the moment, and that often features that are observed may not be able to be unambiguously defined. The interpretation of the data does need to reflect this. Below are several of the main examples we found, but we urge the authors to keep this in mind as they revise the whole manuscript.

We agree with this statement and should have been more conservative with the cryo-ET interpretations. Based on the suggestions from reviewer #2 below, we have made several adjustments to the cryo-ET feature assignments.

a) Assignment of densities: Ribosomes and lipid bilayer are reasonable to assign, because STA in Fig. S3 supports this. Fig. 1 and through the text, eg. lines 120, 126 - proteasomes, PTPs, assigning the outer layer to PTPs, is not justified based on the data. For these, it would be reasonable to speculate in the discussion, it is a reasonable hypothesis, but currently there are no data that directly

support this assignment/interpretation. Statements such as in Line 184 "large-scale remodeling of the PTP layer" - are misleading, and do need to be worded with the appropriate level of certainty, currently it is only a hypothesis that this layer is, in fact, composed of PTPs.

As pointed out, ribosomes and lipid bilayers could be unambiguously assigned, and labels and text sections were kept as in the initial version. While we could identify the typical proteasome side views (see the screengrab of a section from Fig 1 below with the red arrow pointing to a proteasome-like side view), we have changed the labels and text and refer now to "proteasome-like" particles.

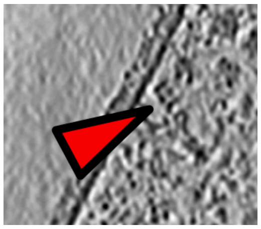

Several previous studies have shown that the outer layer of the PT consists of Polar Tube Proteins which is why we have assigned the outer layer as PTP. We have addressed a similar comment from reviewer #1, points 7 & 8 regarding PTP localization and now provide more background information and additional references on the localization and assignment. We have, however, changed the figure labels from "PTP" to "PT-layer" as our data do not rule out that the surface later may also additionally consist of other components.

b) Definition and classification of Cargo: The authors observe polar tubes with different cargos in them. From the cryo-ET data itself, it is not clear what the cargo is. Based on the timescale of the event, it is unlikely that one would catch a substantial number of tubes in the process of transporting cargo. The data are still valuable, but the authors should take care in how the cargo are interpreted, and what the relationship may be to transport of sporoplasm through the tube.

We agree with this suggestion and add cautionary statements regarding our results, e.g., to indicate a level of uncertainty in the assignment of observed states to a particular temporal order in the germination events (post or pre-sporoplasm translocation – see the response to reviewer #1 points 5 and 6).

We have also adjusted several labels in the figures or throughout the text to indicate the non-unambiguous assignment of some of the observed features ("proteasome-like", "enveloped material", etc.).

Further, an addition (lines 221ff) to the discussion now highlights several aspects of the delicate interpretation that the reviewer mentions: the uncertainty of relative time assignments, as well as possible reasons why we observed different PT states in spite of the rapid nature of the process:

> ***"Firing does not occur simultaneously across all spores, and the implementation of on-grid germination successfully captured heterogeneous states of the PT and sporoplasm***

> ***by immediately vitrifying spores upon inducing firing. It is possible that the mechanical interaction of the PT with the grid support limited tube flexibility, thus slowing down the rapid firing, and trapped otherwise transient states. Two caveats are worth mentioning in relation to this analysis. Firstly, any ordering of the observed structural states onto a timeline is only hypothetical, since cryo-ET does not provide any time resolution. Secondly, we cannot completely rule out that some tomograms represent misfired tubes that fail to fully deliver cargo [25]. However, we made deliberate efforts to avoid imaging such PTs by actively excluding tubes that exhibited bloated appearances and extremely high electron density during the data collection process. "***

c) Time component in interpretation: The authors discuss data as a function of time, for example one section is entitled, "Remodeling of the polar tube protein layer during cargo transport". These data are simply 45 random snapshots of polar tubes, so currently there is no time component in these data. Such a section could be valuable to add to a discussion section, but since it is quite speculative, it would be misleading to a reader for these to be presented as results. Along these lines, Line 118 correlates a "germination phase" with tube thickness. As these experiments have no time component, there is no basis for this correlation. These data can of course be used to generate a hypothesis, which would be appropriate for the discussion section, or clearly indicated that it is speculative (not a direct conclusion from the data presented)

We do not completely agree with the reviewer regarding discussing the cryo-ET data as a function of time. We describe individually observed snapshots of the infection apparatus on an ultrastructural level and correlate the observations based on the most likely scenario to a specific stage in the germination process (e.g., empty sections before or after SP translocation). To prevent confusion, we have removed the section where the correlation with the germination phase was described. Additional changes in lines 109ff should clarify this, e.g., related to the temporal order, and clarify that we observe static snapshots:

> ***"Here, it is worthwhile to note that the PTcargo or PTempty states represent static snapshots after firing and could represent states before or after sporoplasm translocation."***

In addition, we have added statements to the discussion (lines 218ff) to highlight various scenarios to be considered while interpreting our findings. See the response to reviewer #1, point 5, or your comment (b).

d) Line 156-158 - is an overinterpretation of the data, because in our understanding it is currently not known what is in the tubes, and what state they are in. Please re-word.

Line 156 to 158 in the first version of our manuscript reads:

> *"Visualizing polar tube sections in different states allowed us to investigate the ultrastructural organization of the outer layer in empty and filled tubes (**Fig. 1a-e, and Fig. 3a**)."*

Review COMMONS

We are not sure what the reviewer means is an overinterpretation in this statement. We can unambiguously differentiate between empty and filled tubes based on the internal level of electron density. However, to be more specific, we changed "different states" to "cargo-filled" and "empty states" (in lines 149ff), and clarified how it allowed us to investigate the outer layer separately in the two states:

> *"**Inspecting tomograms of PT segments in cargo-filled and empty states** allowed us to **compare** the ultrastructural organization of the outer layer **in empty and filled tubes** (Fig. 1a-e, and Fig. 3a).*

2) One of the main conclusions of the paper is the arrangement of ribosomes in the PT. Yet, these are only observed in 5/45 tomograms. What is the authors' interpretation of this observation? Are they just stuck in the tube in some cases?

Observing identical cargo infrequently, e.g., in only 5/45 tomograms with regularly arranged ribosomes, could be the result of physical, biological, or technical reasons. First, germination and translocation of the SP occurs on a very fast time scale, suggesting it is not very frequent to observe the same section between different imaged PTs. Along those lines, the section imaged is, based on the selected magnification, quite small compared to the entire tube. Based on this, we speculate that the unique observation of the regularly spaced ribosomes in 5/45 samples hints towards a relatively frequent event. As mentioned by the reviewer, it is possible that cargo is stuck within the PT due to technical or biological reasons. For example, it is possible that the interaction of the PT with the EM grid support prevents the release of the SP and traps cargo inside the tube (see also response to reviewer #1, point 5) or that the previously described defective PT could prevent the complete release of the SP.

Similar to our response in comment c, we have updated the discussion section to caution readers about interpreting our findings (see lines 219-330).

3) We request the authors to please provide sufficient information in their methods for reproducibility of their experiments, specifically in these sections:

a) In the germination section, please provide information on reproducibility of the spore preparation, and information on germination rates. Line 104: "with spores consistently displaying high germination efficiencies" - please clarify what "high" means.

Due to unidentified reasons, different batches of produced and purified spores displayed inconsistent germination rates despite the use of similar methods. We have expanded the method section to highlight the observed variation in germination efficiency:

Lines 282:

> ***"In our study, spore preparations for V. necatrix displayed some batch-to-batch variation in germination efficiencies. Hence, a thorough analysis of germination efficiency using alkaline priming [32] was tested out to select a batch for cryoET work."***

Review COMMONS

And lines 287:

> ***"In the batch used for cryo-ET, approximately 80% of spores germinated with the immediate addition of the germination buffer."***

b) Light microscopy: please specify rates of incomplete and complete germination, how this was evaluated, how many events were analyzed, and any differences between complete (sporoplasm visible) or incomplete (sporoplasm not visible) germination.

The reviewer brings up an interesting point. We did indeed notice incomplete germination events where the sporoplasm was not ejected from the PT. This accounted for 5 of the 58 monitored germination events. These events were excluded from the length and velocity analyses, which were intended to study the complete and unabrogated germination of the polar tube. However, these partial germinations are most intriguing because they lack the typical refractory shortening that occurs after sporoplasm extrusion, and instead remain close to their maximal length. Although we find this interesting, we refrained from discussing these quirks in the text due to inconsistencies in incomplete germination rates, and a lack of evidence as to the physiological relevance of this observation. Similar to our above comment in point 3A, the rate of incomplete germination varies significantly from batch to batch of purified microsporidia, and the origins of this variation are currently unclear. However, as this information may eventually prove valuable, we have added a comment to the methods (lines 288ff) clarifying incomplete germination rates and our observation that these tubes did not display refracted lengths after sporoplasm extrusion.

> ***"In approximately 9% of the monitored germination events, sporoplasms were not ejected from PTs and tube lengths did not decrease after reaching their maximal length. As the goal of this study was to describe and characterize successful germination conditions, these incomplete germination events were excluded from all analyses."***

c) Line 325: please provide detailed information on CNN-based picking and segmentation, for example, parameters used for optimization

We have expanded the method section (lines 325ff) and are now providing additional details on picking and segmentation:

> ***"For segmentation, default pipelines for importing and preprocessing tomograms were used. Areas of interest were picked with a box size of 64, and a pen size of 1 was used for annotating the particles. The ratio of the number of particles of interest to negative reference was roughly 1:20, and all network training parameters were kept unchanged. All particles of interest were segmented using separate CNN-based models, and the output from each model was grouped using Segger [72], and visualized in ChimeraX. The binned, denoised tomogram projections were also used to manually measure membrane thickness and inter-ribosome distances using IMOD and Dynamo. For measurements on PT outer layers, 10 tomograms each from PTempty and PTcargo were surveyed uniformly across their length to calculate the mean thickness for each tomogram (n = 10, for each tomogram) (Figure 3c, Supplementary Fig. 5)."***

Review COMMONS

d) Line 330: please specify number of tomograms
See response to f)
e) Line 331: please specify how manual alignment and particle centering was achieved
See response to f)
f) Line 332: please provide information on template-matching options / thresholds used

As a response to comments d) to f) we have updated the sub-tomogram averaging method section (lines 336ff) accordingly with the missing information:

> *"Subtomogram averaging was carried out as schematically indicated in (**Supplementary Fig. 3**). Around 150 particles were manually picked and extracted from two 4-times-binned tomograms using Dynamo [73]. The resulting particles were* ***manually centered and aligned by visual inspection in the dgallery function in Dynamo. Manually aligned particles were then averaged to generate an unaligned average.*** *Subsequently, the inbuilt template-matching function in Dynamo was used to pick particles using a low-pass filtered initial model. Post template matching,* ***picks were selected from individual tomograms based on cross-correlation scores upon manual inspection."***

4) Supporting Fig 2c: we found it confusing to understand how Pempty is defined, it does not look empty in some cases, and the 3 shown look very different. On what basis is the tube labeled "empty"? The definition provided in line 131 does not seem to match the figures.

Please see the response to a similar comment from reviewer 1, point 13.

Minor comments:

5) Fig. 1: The lipid bi-layer in parts a to e seems different, and we found this confusing. Is the pink label in A not pointing to the correct layer?

It is correct that the pink label points to the lipid bilayer. When internal membranous organelles pass through the tube, multiple bilayers might be present. In some cases, potentially panel a or panel d, the bilayer of these internal structures or other membranous vesicles, are close to the outer membrane, which could contribute to the confusion in defining the outer membrane layer. We have labeled clearly identifiable internal lipid bilayers with blue arrows.

The corresponding segmentation is also confusing - does the lipid bilayer not go all the way around the tube? This would be important to clarify, since a lipid bilayer is one of the major components of the tube.

It is very reasonable to assume that the membrane indeed goes all the way around the tube. However, the inherent anisotropicity of cryo-electron tomography makes the membrane less well-defined at the top and bottom. We thus, as is customary in cryo-ET papers, chose to segment only the well-defined sides of the membrane, which also gives the added benefit of not obscuring the tube contents.

6) The following publications have shown cryo-ET of the polar tube, and should be referenced appropriately in the introduction, as well as during interpretation: 1) BioRxiv,https://doi.org/10.1101/2023.05.01.538940 Figure 1 and 2) PMID: 31332877. The second is referenced but should be mentioned around line 70 in the introduction

We have added the reference of Qing Lv et al and Takvorian et al, 2020 to the introduction.

7) In the introduction, it would be helpful if the authors mention something about their microsporidia species of interest, and reason for choosing to study this species.

Vairimorpha necatrix is an ideal model organism for cryo-ET studies, as multiple cellular macromolecules from this organism have been solved. The structures solved from this organism are cited in the manuscript.

8) Fig. S1C - please show individual data points

In response to reviewer #1, point 3, we have updated **Supplementary Figure 1c** with an additional plot showing the final length of the polar tube and the level of PT retraction after reaching maximal length. In addition, we now show all individual data points in all plots. See the updated Figure above (reviewer #1, point 3).

9) Line 100 - the data presented do not show deformation of the cargo, so please reword to reflect the data being discussed

Changed the section (lines 87ff) accordingly and added the reference to Chang et al, 2023.

> *"Akin to tube remodeling events, the sporoplasm* ***also likely transforms from a restricted*** *spore state to extended conformation during extrusion* ***[30]****, into a* ***spherical*** *shape upon emergence (****Supplementary Fig. 1a****), and these events may impose immediate reorganization of subcellular structures."*

10) Line 140: could the authors please outline how they confirmed that the handedness of the reconstructed tomogram is correct?

The same concern was raised by Reviewer 1, which is why we have removed the definition of exact handedness. See also our response to reviewer 1, point 1.

11) Line 250: re-word to ensure that appropriate credit is given to previous work in the field; the large-scale rearrangement of the polar tube has been observed for many decades

We have adjusted the sentence on line 248ff and referenced the comprehensive book chapter from Jaroenlak et al.2022:

> *"Our work **confirms previous observations (Jaroenlak et al, 2022)** that the polar tube undergoes large-scale structural reorganization during germination."*

12) Line 352: out of curiosity, why could resolution not be determined for PTcargo?

We hypothesize that the flexibility of the PT layer and the presence of an electron-dense membrane may contribute to the incorrect estimation of resolution for the PTcargo. This hypothesis is supported by the dissonance in resolution estimation by the Dynamo package during refinement runs and by the EMDB submission pipeline. Given this difference between the estimates for the STA map, we report these estimates as undetermined.

13) Fig S2a: diagram of the polar tube within the spore shows the polar tube with opposite handedness to what has been previously determined

Thank you for spotting this mistake. In response to a similar comment from reviewer #1, we have corrected the orientation of the coils in the spore schematic drawings in Fig 4 and Supplementary Figure 2a.

14) Supporting table 1 - is missing frames per movie and which mode data were collected in

The frames per movie and data collection mode have been added.

15) Fig 2b: We did not follow the rationale for the 3 colors of ribosomes

The different colors should serve as a visual guide for the reader and show the spiral arrangement of the ribosomes along the tube more clearly. The beige-colored ribosomes also follow the spirals but are not colored as parallel strands as they are not visible from the xy-plane.

To further clarify, we have updated the figure legend accordingly:

> *"(**a**) Slices through a cryo-tomogram and the corresponding neural network aided 3D segmentation (**b**) of a polar tube filled with clustered ribosome arrays. The segmentation presents ribosomes in yellow, green, or beige. **The yellow and green colored particles indicate the front view from the xy-plane, while the beige-colored particles cluster at the distal end of the same plane**."*

16) Sup Fig 3a: please specify in legend and/or workflow software packages used in panel (a)

We have utilized the Dynamo package throughout our STA processing. As the reviewers suggested, we added this information to the legend in **Supplementary Figure 3**. Accordingly, we have elaborated on our STA methodology in the methods section.

17) Fig 2e: it is unclear whether averages presented are from 1 tomogram, or all tomograms where that pattern is visible - Is the measurement coming from all 5 tomograms?

The legend corresponding to the schematic representation in Figure 2e has been updated to clarify that measurements were taken from 3 out of 5 tomograms.

18) Fig 3 c-e: it is unclear how many tomograms were used for these averages. Was 1 STA per tomogram performed, or 1 STA per type of PT?

The STA processing scheme, including the number of used tomograms, is indicated in Supplementary Figure 3, and we have also expanded on the same in our methods section.

For the thickness measurement in c, all individual measured data points are now added to the supplement in a new **Supplementary Figure 5** (below), and the method section has been updated to describe how boundaries were defined. See our response to reviewer #1, point 12.

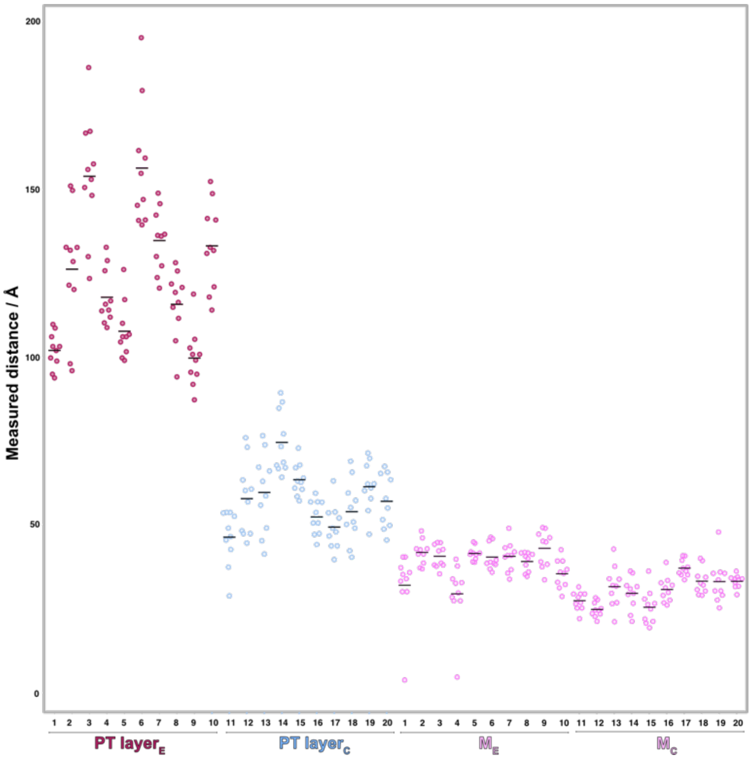

"Supplementary Figure 5: Individual data points for PTP Layer measurements: *A plot showing the distribution of individual data points measured for the thickness of various features of PTempty and PTcargo tubes (10 tubes each). Thickness was measured on tomographic projections along the length of the tubes and mean value for each measurement is indicated for each tube. The individual data points were utilized to derive measurements shown in Figure 3."*

Reviewer #2 (Significance (Required)):

Overall, this paper provides interesting new insights into knowledge of the microsporidian polar

tube. We thank the authors for making this paper available to the community on BioRixiv, and we summarize a few main comments below, which we hope will be helpful in preparing a revised version of the manuscript. There is a substantial advance in applying cryo-ET to studying the polar tube of microsporidian parasites. The audience this will be interesting to are those studying microsporidian parasites.

Thank you again for carefully reviewing our manuscript, the constructive comments, and for acknowledging our BioRxiv deposition.

Reviewer #3 (Evidence, reproducibility and clarity (Required)):

This paper by Sharma et al describes ultrastructural changes in the polar tube (PT) of the microsporidian species Varimorpha necatrix upon PT firing. The relationship between cargo transport and the diameter of the PT, as well as the thickness of the PTP coat, are investigated. Moreover, low-resolution sub-tomogram averaging (STA) reveals that ribosome dimers occasionally arrange into spiral-like arrays on the inner surface of the bilayer lining the PTP coat. The data are well presented and, in most parts, appropriately interpreted. I have the following comments that I suggest should be addressed.

We thank the reviewer for their positive remarks on the data presentation and interpretation.

Major:

1. The authors suggest that the ribosomes in the arrays are dimers. Yet, figure 2c only shows a STA map of a 70S particle. A map of the dimer should be included to support this significant message of the paper.

We agree with the reviewer's observation about the importance of a dimer map to our work. For this, we have attained an STA map of the dimer and updated it in Figure 2f. Technical limitations of low particle number (around 370 particles) and specific orientation of dimer particles concerning imaging direction resulted in a low-resolution map that agrees well with the one observed in McLaren et. al. *Nature Microbiology* 8, 1834:1845 (2023). We have added the low-resolution dimer map to the main manuscript as **Fig. 2f**.

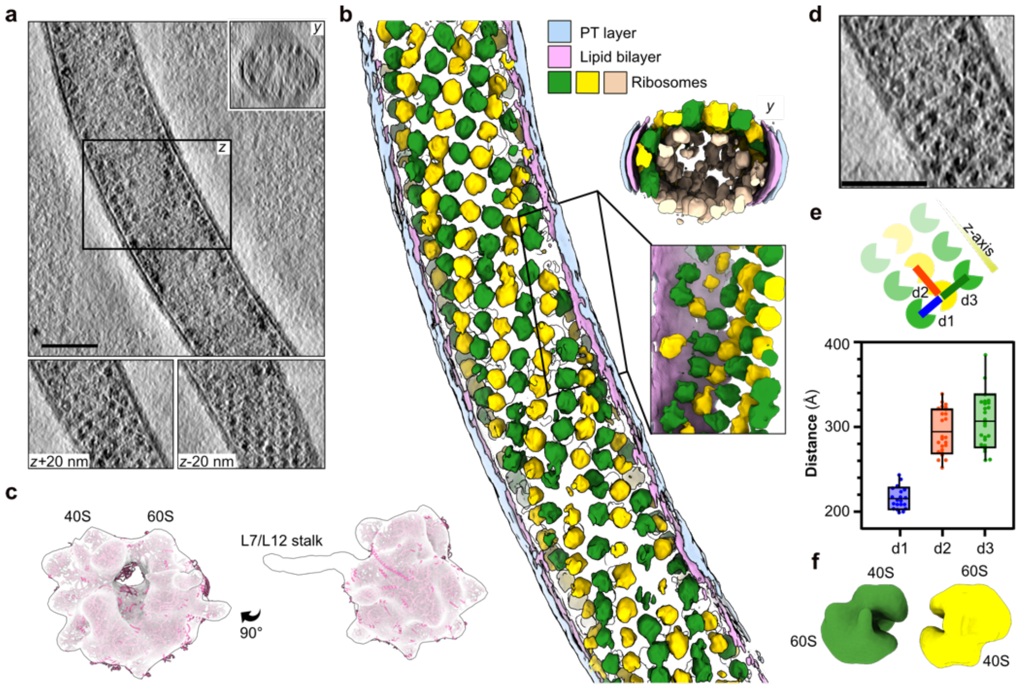

Fig. 2 ……" (f) A subtomogram average of ribosome dimer particles is shown lowpass filtered to a resolution of 50 Å, and colored as in (b)."

2. Do the authors see any 70S particles and if so, how common are they? 3D classification would clarify this.

We observe ribosome monomers in our tomograms, as depicted in Fig 1b. In fact, a 70S particle seems to be the norm in sporoplasm-filled tubes. A subset of the cargo-containing tubes, as represented in **Fig. 2** and **Supplementary Figure 2**, contain the spirally arranged ribosome dimers. The difference in the distribution of the prevalence of these states can also be seen in particles utilized for subtomogram averaging experiments. For monomers, we could extract ~3000 monomers while we could get only <400 ribosome-dimer-like particles. Here, the monomer picks are expected to include particles corresponding to the dimer-like particles as well.

3. The authors put a lot of emphasis on the finding of array-like ribosomes within PTs. However, these appear to be present in only a minority of the cases. Moreover, similar ribosome arrays have previously not been seen in other microsporidian PTs. This raises the question of how significant these arrays are. Do they only occur in some microsporidia, or only at certain time points? This should be more clearly discussed, for example in lines 232 - 233. Also, do the authors suggest that this is a specific organisation or just a matter of close packing?

Observing the ribosome arrays in a fraction of tomograms could result from a variety of physical, biological, or technical reasons. First, germination and translocation of the SP occurs on a very fast time scale, suggesting it is not very frequent to observe the same section between different imaged PTs. Along those lines, based on the selected magnification, the section imaged is quite small compared to the entire tube. Based on this, the unique observation of the regularly spaced ribosomes in more than one of a relatively small number of samples hints towards a relatively frequent event. It is further possible that the cargo is stuck due to technical or biological reasons. For example, it is possible that the interaction of the PT with the EM grid support prevents the release of the SP and traps cargo inside the tube (see also response to reviewer #2, point 2) or that the previously described defective PT could prevent the complete release of the SP.

Accordingly, to provide a thorough view of the observed ribosome spiral, we added additional segmentation in **Supplementary Figure 2** and cautionary statements in the text (lines 218ff) to highlight that the observed sporoplasm states could arise from various biological and experimental factors.

4. As the arrays are only occasionally observed, the statement that ribosomes are transported through PTs in a spiral-like fashion should also be toned down in the abstract and throughout the manuscript. The fact that the arrays are only seen sometimes, makes the finding even more interesting, as it may infer a dynamic reorganisation process.

We agree with the reviewer's comment and have incorporated the suggested changes in the abstract. Further, to highlight the dynamic reorganization of ribosomes during cargo transport, we have added the following statement to the discussion section (lines 235ff):

> ***"Here, the occurrence of free flowing (Fig. 1b), as well as spirally packed ribosomes (Fig. 1c and 2) in germinated tubes likely represents two dynamic states that may interconvert during host invasion."***

5. The ribosome arrays appear to co-localise with the membrane. If this is the case, does the membrane show up in their STA? If so, it would be essential to show this.

See response to comment 6.

6. How do the ribosomes in the arrays differ from the free-floating ones? Are the latter not associated with the membrane, while the former are not? Can differences be visualised through 3D classification?

The reviewer raises a very interesting point about the colocalization or possible association of ribosomes with the PT membrane in comments 5 and 6. A membrane association would be reasonable for particles clustering along a membrane, which we have investigated inconclusively with STA. In all our STA trials for the membrane-proximal particles, the presence of the PT surface during subtomogram alignment substantially worsened the alignment of the ribosomes to the point where no specific features were discernible, suggesting large variability in their orientation with respect to the membrane. This was further complicated by variations in membrane curvature depending on the region from which the ribosomes were extracted. In these attempts, we did not observe any density connecting ribosomes to the membrane.

7. The difference in the PTP coat in empty vs. filled PTs are very interesting. Can the authors clarify how this was measured and mention the number of measurements, mean, and standard deviation in the main text? Line plots would help substantiate the measurements.

We have added experimental details about the measurement of PT layers to the methods sections (lines 330ff). Further, we have also added plots from individual measurements taken along the length of the PT as a new Supplementary Figure 5.

The method section now includes a description of the distance and thickness measurements in paragraph:

"Tilt series processing, tomogram generation, segmentation, ***and distance measurements"***

Please also, see the response to a similar suggestion from reviewer 1, point 12.

8. Do the authors observe any differences in the regularity of the array? This could be assessed by investigating power spectra of tomograms of STAs.

We observe differences in the arrangement of ribosome clusters in the spirals. The difference is visible in the segmented tomograms. We have added two additional tomogram segmentations to **Supplementary Figure 2e** and **2f**.

9. How do the authors suggest such large changes in thickness come about? Is the PT coat "bunched up", as the PT compresses and stretched out, as the PT extends?

Based on the STA volumes presented in Figure 3, we speculate that the lipid bilayer provides a fluid membranous base for the PT coat to stretch or relax during firing and sporoplasm discharge. We suggest this in the schematic drawing in **Fig. 3e**. However, high-resolution structural data of the PT layer and its interaction with the membrane, is necessary to substantiate this hypothesis.

10. How often are each of the described PT stages seen as a percentage of all data? Are some observed more often than others or is the distribution equal?

As our tomograms were collected based on visual inspection of elongated PTs, we believe that drawing any conclusions about the distribution of PT states may not be accurate. In the absence of an unbiased quantitative analysis, we prefer not to include these distributions. If the reviewer still wishes us to include this information in spite of our justification for not doing so, we can add it in a tabular form.

11. Line 125. How do the authors know that they observe nuclei? Can they identify nuclear envelopes? Are nuclear pores evident?

No, we do not observe nuclear envelopes or pores in the data presented here. Thus, without any direct observations of such particles, we have withdrawn this claim from our text. Please also see a similar comment made by reviewer #1 under point 8.

12. Line 168 - 169: How many measurements were taken from each state? What was the mean and SD for membranes and coats? This will be interesting, especially, as the thickness of the PT coat can vary along the length of one PT.

We indeed observe some variation in PT layer thickness along its length. Hence, we sampled the entire length of the tube with uniformly distributed points to calculate the mean thickness. We have updated the method section "*Tilt series processing, tomogram generation, segmentation, and distance measurements*" to include more information on how the thickness was measured (lines 330ff):

> ***"The binned, denoised tomogram projections were also used to manually measure membrane thickness and inter-ribosome distances using IMOD and Dynamo. For measurements on PT outer layers, 10 tomograms each from PTempty and PTcargo were surveyed uniformly across their length (n = 10, for each tomogram), to calculate the mean thickness for each tomogram representing each measurement (Figure 3c). "***

For completeness, we have now included all individual thickness measurements of the PT layer in **Supplementary Figure 5**.

Minor:

Review COMMONS

1. Line 102 "optimal conditions" sounds obscure, please briefly mention what these are.

We have updated the methods section with the rationale and details on the alkaline priming conditions used for spore germination.

2. Line 119. Are membrane-less PTs ever seen?

No, we did not observe PTs without a membrane. We have added a statement on line 114:

> ***"Membrane-less PTs were not observed."***

3. Line 156, the word "remodelling" may be too specific, considering that only differences in thickness were measured.

We changed the section title to:

> ***"Adaptation in the polar tube layer during cargo translocation"***

4. Line157: "Visualising PT sections ...." Sections sounds like physical cryosections were investigated. Perhaps better: "Inspecting tomograms of PT segments in different states..."

We have modified the text in accordance with your suggestion on line 149.

5. Line 161: "subtomogram averaging particles picked on the tube wall from both states" better: "subtomogram averaging of the tube wall from both states"

We agree and have updated the sentence (line 153).

6. Line 162: delete "it be"

Changed "it be" to "it is"

7. Line 201: Is organ the right word here?

We have replaced organ with organelle on line 192.

8. Fig 2: Increase transparency to reveal the atomic model in C more clearly.

We have updated the figure as per the suggestion.

9. Fig 2b. I suspect the beige ribosomes are ones that do not follow the array? If so, can you please clearly state it? Also, are these dimers too? And can you tell if they are different?

Review COMMONS

The yellow and green colors should serve as a visual guide for the reader and show the spiral arrangement of the ribosomes when seen from the xy plane. The coloring scheme is chosen for purely aesthetic purposes. As per the segmentation analysis, the beige-colored ribosomes also form the spiral at the distal end of the xy plane. For clarification, we included here the following image showing the distal view of the ribosome-filled PT:

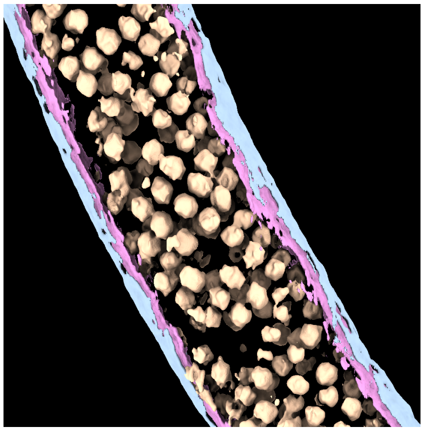

To further clarify the coloring scheme, we have updated the figure legend accordingly:

> "(**a**) Slices through a cryo-tomogram and the corresponding neural network aided 3D segmentation (**b**) of a polar tube filled with clustered ribosome arrays. The segmentation presents ribosomes in yellow, green, or beige. ***The coloring scheme is chosen for aesthetic purposes, where yellow and green colored particles are arranged on the front view of the xy-plane while beige-colored particles cluster at the distal end of the same plane***."

10. Fig. 3e: The diagonal lines in the schematic infer that the data provide some level of insight into the PT lattice structure. As this is not the case, it would be better to remove these lines.

We have removed the diagonal lines in Fig. 3e. See the updated Figure 3 as part of our response to reviewer #1, point 14.

13. A flow chart highlighting the sub-tomogram averaging workflows employed should be included.

We have updated the STA workflow in the method section and **Supplementary Figure 3**.

Reviewer #3 (Significance (Required)):

This paper advances our knowledge of the microsporidian polar tube with regard to its structure, dynamics, and transported content. Ribosome arrays have not been described before in extended PTs, so this is an interesting discovery, which adds to the complexity of ribosome regulation in microsporidia.

Full Revision

Strengths are the novelty of the findings, in particular the ribosome arrays, PT dynamics, and PT composition.

As a weakness, I feel that the tomography data could have been analysed in more depth. For example, at least a low-resolution map of the ribosome dimer would be important to show that the ribosomes in the arrays are indeed dimers. In addition, 3D classification would be useful to understand, if all ribosomes occur as dimers or only a fraction.

The paper is clearly written and well presented and thus suitable for a wider audience, including researchers studying microsporidia, infection biology, host-pathogen interactions, and ribosome biology.

We wish to thank the reviewer for the careful assessment, the very productive review, and the positive comments on the data presentation and writing. We also appreciate the reviewer's specific comments about additional data and believe this has helped to strengthen our story. We hope that we could address all concerns in the revised version.
