# Supplementary material for: Ultrastructural insights into the microsporidian infection apparatus reveal the kinetics and morphological transitions of polar tube and cargo during host cell invasion
Source: PLoS Biol. 2024 Feb 29;22(2):e3002533. doi: 10.1371/journal.pbio.3002533 (PMC10931468; doi:10.1371/journal.pbio.3002533)
Supplement: Attachment — Submitted filename: Sharma_et_al_Response_to_reviewers.pdf [file pbio.3002533.s014.pdf]

Responses to Reviewers' remarks:

We appreciate the reviewers providing constructive feedback on our study and are pleased to hear their positive feedback on the revised manuscript. Below, we have addressed the remaining minor open points with our responses in blue.

Reviewer #1 (Damian Ekiert, signs review):

1. While the authors have mostly responded adequately to my comments, I still object to equating the apparently proteinaceous layer that they observe by cryo-ET with the polar tube proteins (PTPs). This was Major Comment 7 from Reviewer 1, and also Major Comment 1a from Reviewer 2, so clearly this was a conceptual leap that two reviewers objected strongly to. As I said in my original review, it is a reasonable hypothesis that can be discussed, but it is an unsupported and speculative claim. However, while several PTPs have been shown to localize along the length of the PT by IF, the PTP is only ~100 nm is diameter and necessarily diffraction limited. Light microscopy can't readily distinguish what is inside versus outside. Previous EM has suggested an outer protein layer, but what it is made of one can only guess. Additionally, the localization of PTPs along the PT doesn't necessarily mean that they are structural components, or even major components. For example, the layer the authors observed by cryoET may be formed predominantly by a protein that remains to be discovered. The changes to the manuscript don't adequately resolve this problem. For example, lns 111-113 of the revised manuscript state, "...the tube wall, which is composed of a lipid bilayer (pink arrows, Fig. 1 & Supplementary Fig. 2), flanked by an outer layer of polar tube proteins (light blue and magenta arrows, Fig. 1 & Supplementary Fig. 2)." This assignment of the outer layer as consisting of PTPs is not supported by the data and should be removed. Calling it the "Polar tube layer" or "PT layer" also seems to suggest equivalence. I suggest sticking closer to the data and what is known, and referring to it as the "outer layer", "protein layer", or something similar to avoid misleading readers.

We have rephrased text reference to the outer layer of the polar tube now as “protein layer”. These changes have been incorporated throughout the text and the figures.

Reviewer #2: We thank the authors for their extensive and thoughtful responses, and we found the manuscript to be substantially improved. We feel it is a very nice and timely contribution to the field.

We had a few outstanding issues that we would like to point out.

We do disagree with assigning the "PTP layer". This is not supported by data. We feel it is fine to speculate this in the discussion, with a model, which would be very reasonable, but it is confusing for the field when strong conclusions are made which are not adequately supported by data, and this can lead to incorrect assumptions being propagated indefinitely, which is damaging to the field.

Please see our response to a similar comment from reviewer #1.

We do feel it is quite confusing to refer to "Empty" tubes that are not empty.

We agree with the reviewer’s suggestion, and we have now updated the description of PTempty with further clarification in the text and figures.

E.g. around line 126:

> “Notably, macromolecular complexes were completely missing from *PTempty*, which mostly housed electron-lucent vesicles”

Or supplementary Figure 2 legend:

> ”**(c**) Representative tomograms of *PTempty*, or polar tubes filled with electron-lucent material or completely devoid of cellular cargo.”

In the methods, we request the authors to please clarify what CNN architecture was used and where it comes from.

We have added the following details to the methods section (Tilt series processing, tomogram generation, segmentation, and distance measurements) for additional details on the CNN architecture:

> “EMAN2’s inbuilt, default 4-layer CNN architecture was utilized for training networks for annotating features on the tomogram.”

Just to clarify, our comment regarding whether the lipid bilayer goes all the way around was not referring to the top/bottom anisotropy, but rather to the holes in the side. We were curious as to whether the authors believe there are holes or not, and to please clarify their interpretation for the reader.

We do not think there are holes in the membranes, and we believe these arise from technical limitations while annotating the features. We have clarified this by adding the following section to the main text on lines 119ff:

> “Membrane-less PTs were not observed, and the PT membrane appeared continuous in all raw tomograms (small discontinuities in the membrane auto-segmentation likely being the result of mis-annotation due to limited signal-to-noise ratio).”

Reviewer #3: Through their revisions, the authors were able to significantly improve their manuscript and I have no reservations against its publication in PLOS Biology.

I would like to thank the authors for addressing all of my comments so thoroughly and congratulate them to this fine paper.

We thank you for your positive evaluation of our revisions.
